# Aging and Comorbidities in Acute Pancreatitis I: A Meta-Analysis and Systematic Review Based on 194,702 Patients

**DOI:** 10.3389/fphys.2019.00328

**Published:** 2019-04-02

**Authors:** Katalin Márta, Alina-Marilena Lazarescu, Nelli Farkas, Péter Mátrai, Irina Cazacu, Máté Ottóffy, Tamás Habon, Bálint Erőss, Àron Vincze, Gábor Veres, László Czakó, Patrícia Sarlós, Zoltán Rakonczay, Péter Hegyi

**Affiliations:** ^1^Institute for Translational Medicine, University of Pécs Medical School, Pécs, Hungary; ^2^János Szentágothai Research Center, University of Pécs, Pécs, Hungary; ^3^County Emergency Clinical Hospital of Timisoara, Clinic II Pediatrics, Timisoara, Romania; ^4^Institute of Bioanalysis, University of Pécs Medical School, Pécs, Hungary; ^5^Research Center of Gastroenterology and Hepatology, Craiova, Romania; ^6^Division of Cardiology, First Department of Medicine, University of Pécs Medical School, Pécs, Hungary; ^7^First Department of Medicine, University of Pécs Medical School, Pécs, Hungary; ^8^Department of Pediatrics, University of Debrecen, Debrecen, Hungary; ^9^First Department of Medicine, University of Szeged, Szeged, Hungary; ^10^Department of Pathophysiology, University of Szeged, Szeged, Hungary; ^11^MTA–SZTE Momentum Translational Gastroenterology Research Group, University of Szeged, Szeged, Hungary

**Keywords:** acute pancreatitis, aging, mortality, severity, co-morbidity

## Abstract

**Background:** Acute pancreatitis (AP) is one of the most common cause of hospitalization among gastrointestinal diseases worldwide. Although most of the cases are mild, approximately 10–20% of patients develop a severe course of disease with higher mortality rate. Scoring systems consider age as a risk factor of mortality and severity (BISAP; >60 years, JPN>70 years, RANSON; >55 years, APACHE II >45 years). If there is a correlation between aging and the clinical features of AP, how does age influence mortality and severity?

**Aim:** This study aimed to systematically review the effects of aging on AP.

**Methods:** A comprehensive systematic literature search was conducted in the Embase, Cochrane, and Pubmed databases. A meta-analysis was performed using the preferred reporting items for systematic review and meta-analysis statement (PRISMA). A total of 1,100 articles were found. After removing duplicates and articles containing insufficient or irrelevant data, 33 publications involving 194,702 AP patients were analyzed. Seven age categories were determined and several mathematical models, including conventional mathematical methods (linear regression), meta-analyses (random effect model and heterogeneity tests), meta-regression, funnel plot and Egger's test for publication bias were performed. Quality assessment was conducted using the modified Newcastle–Ottawa scale. The meta-analysis was registered in the PROSPERO database (CRD42017079253).

**Results:** Aging greatly influences the outcome of AP. There was a low severe AP incidence in patients under 30 (1.6%); however, the incidence of severe AP showed a continuous, linear increase between 20 and 70 (0.193%/year) of up to 9.6%. The mortality rate was 0.9% in patients under 20 and demonstrated a continuous linear elevation until 59, however from this age the mortality rate started elevating with 9 times higher rate until the age of 70. The mortality rate between 20 and 59 grew 0.086%/year and 0.765%/year between 59 and 70. Overall, patients above 70 had a 19 times higher mortality rate than patients under 20. The mortality rate rising with age was confirmed by meta-regression (coefficient: 0.037 CI: 0.006–0.068, *p* = 0.022; adjusted r^2^: 13.8%), and severity also (coefficient: 0.035 CI: 0.019–0.052, *p* < 0.001; adjusted r^2^: 31.6%).

**Conclusion:** Our analysis shows a likelihood of severe pancreatitis, as well as, pancreatitis-associated mortality is more common with advanced age. Importantly, the rapid elevation of mortality above the age of 59 suggests the involvement of additional deteriorating factors such as co-morbidity in elderly.

## Introduction

### Rationale

**Figure 1:**
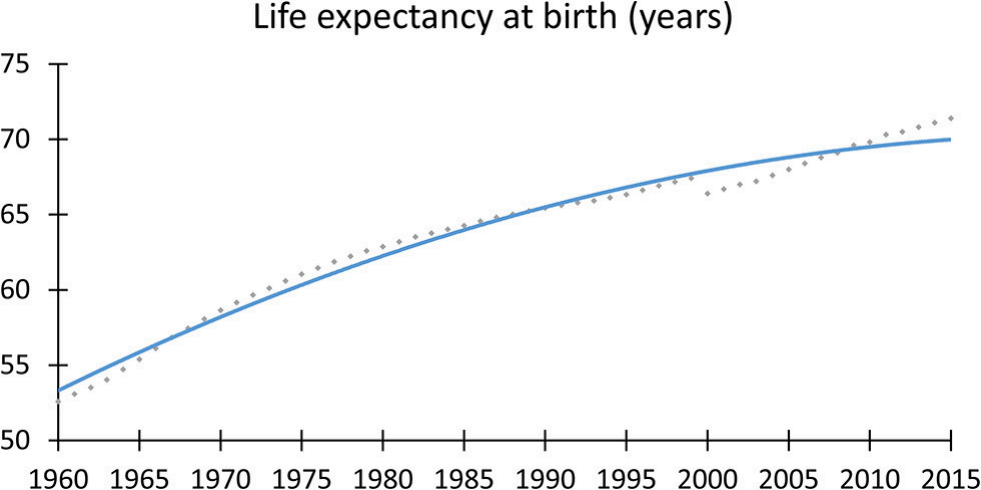
Life expectancy at birth. There is a steadily rising average life expectancy at birth. It has dramatically risen by 16 years (from 55.4 to 71.4 y) in the last half century. Data sources: between 1960 and 1999, World Bank; between 2000–2015, WHO.

Life expectancy has dramatically risen by 16 years (from 55.4 years to 71.4 years) in the last half century, causing a number of changes and challenges to economies and healthcare systems (Figure 1). Needless to say, healthcare professionals should focus more intensively on the effects of aging on the course and outcome of diseases.

Acute pancreatitis (AP) is one of the most challenging gastrointestinal disorders: (1) its development is not fully understood (Sahin-Toth and Hegyi, 2017) and it has no specific therapy (Hegyi and Petersen, 2013); (2) its incidence rate is continuously increasing (Peery et al., 2015); and (3) it has an unacceptably high mortality (Parniczky et al., 2016). Unfortunately, gastrointestinal scientists are devoting ever less attention to AP (Szentesi et al., 2016). One of the best examples of this is that mathematical analysis on the effects of aging on many diseases, such as neurophysiological and liver disorders, have been performed (Mizuguchi et al., 2015) but no systematically collected information is available on AP.

### Objectives

Age is used as a predictive marker in different scoring systems for AP (Table 1). These scoring systems show a great variety in the age group: in the (i) Bedside Index for Severity in Acute Pancreatitis score (BISAP) (Wu et al., 2008), the topmost risk of age is above 60; (ii) in BALI (BUN, Age, LDH, IL-6), it is over 65 (Spitzer et al., 2006); (iii) in the Simplified Acute Physiology Score (SAPS II), it is >40 (Legall et al., 1993); (iv) in Ranson score, it is above 55 (Blamey et al., 1984); (v) in Acute Physiology and Chronic Health Evaluation (APACHE II), it is over 45 (Wagner and Draper, 1984); and (vi) in the Japanese Severity Score (JNP), it is >70 (Hirota et al., 2006). The wide range of age limits suggests that a low number of patients, a selection bias and/or a mathematical inaccuracy could have occurred.

**Table 1:** Characteristics of the scoring systems.

**Score system**	**Publ. (year)**	**Outcome**	**Time at measurement**	**Age cutoff**	**Patient enrolment**	**LEB**	**Age**	
							**Med**.	**Mean**
Ranson	1974	Severity	48 h	55	1971–1975	60.12		42 50
APACHE II	1982	Severity	24 h	45	1979–1981	62.9	–	
SASP II	1993	Mortality	last 24 h	40	1991	65.6		57.2
JPN	2002	Severity	–	70	1995–1998	66.75	–	
BALI	2006	Mortality	48 h	65	–	–		61 ± 16
BISAP	2008	Morality	24 h	60	2000–2001	66.55	53	

*There is a slight elevation in the age of enrolled patients and cut-off values (LEB: Life expectancy at birth). Ranson (Blamey et al., 1984); APACHEII–Acute physiology and chronic health evaluation (Wagner and Draper, 1984); SAPS II–Simplified Acute Physiology Score (Legall et al., 1993); JNP–Japanese Severity Score (Hirota et al., 2006); BALI–BUN, Age, LDH, IL-6 (Spitzer et al., 2006); BISAP–Bedside Index for Severity in Acute Pancreatitis (Wu et al., 2008)*.

### Research Question

In order to minimize these distorting factors, we aimed to (i) comprehensively search and select articles in which all AP cases have been included and (ii) use several mathematical models to understand the effects of aging on the outcome of AP.

## Methods

### Study Design, Participants, Interventions, Comparators

The meta-analysis was performed using the preferred reporting items for systematic review and meta-analysis statement (PRISMA) (Moher et al., 2009). We used the classical PICO format to form a question applicable for search in databases: P: acute pancreatitis; I and C: different age categories [under 20 (U20), 20–29, 30–39, 40–49, 50–59, 60–69, and above 70 (A70)]; O: mortality and severity. In order to provide the highest level of quality, the meta-analysis was registered with the PROSPERO registry (CRD42017079253).

### Search Strategy

A search was performed in three databases (Embase, PubMed and Cochrane) in January 2017 using the following terms: PubMed: {acute[All Fields] AND (“pancreatitis“[MeSH Terms] OR “pancreatitis”[All Fields])} AND {cohort[All Fields] OR (“clinical trial”[Publication Type] OR “clinical trials as topic”[MeSH Terms] OR “clinical trial”[All Fields])} AND (“Age”[Journal] OR “age”[All Fields] OR “Age (Omaha)”[Journal] OR “age”[All Fields] OR “Age (Dordr)”[Journal] OR “age”[All Fields] OR “Adv Genet Eng”[Journal] OR “age”[All Fields]) Embase: acute pancreatitis and (cohort or clinical trial) and age; and Cochrane: acute AND pancreatitis AND (cohort OR clinical) AND trial AND age.

### Data Sources, Study Selection, and Data Extraction

**Figure 2:**
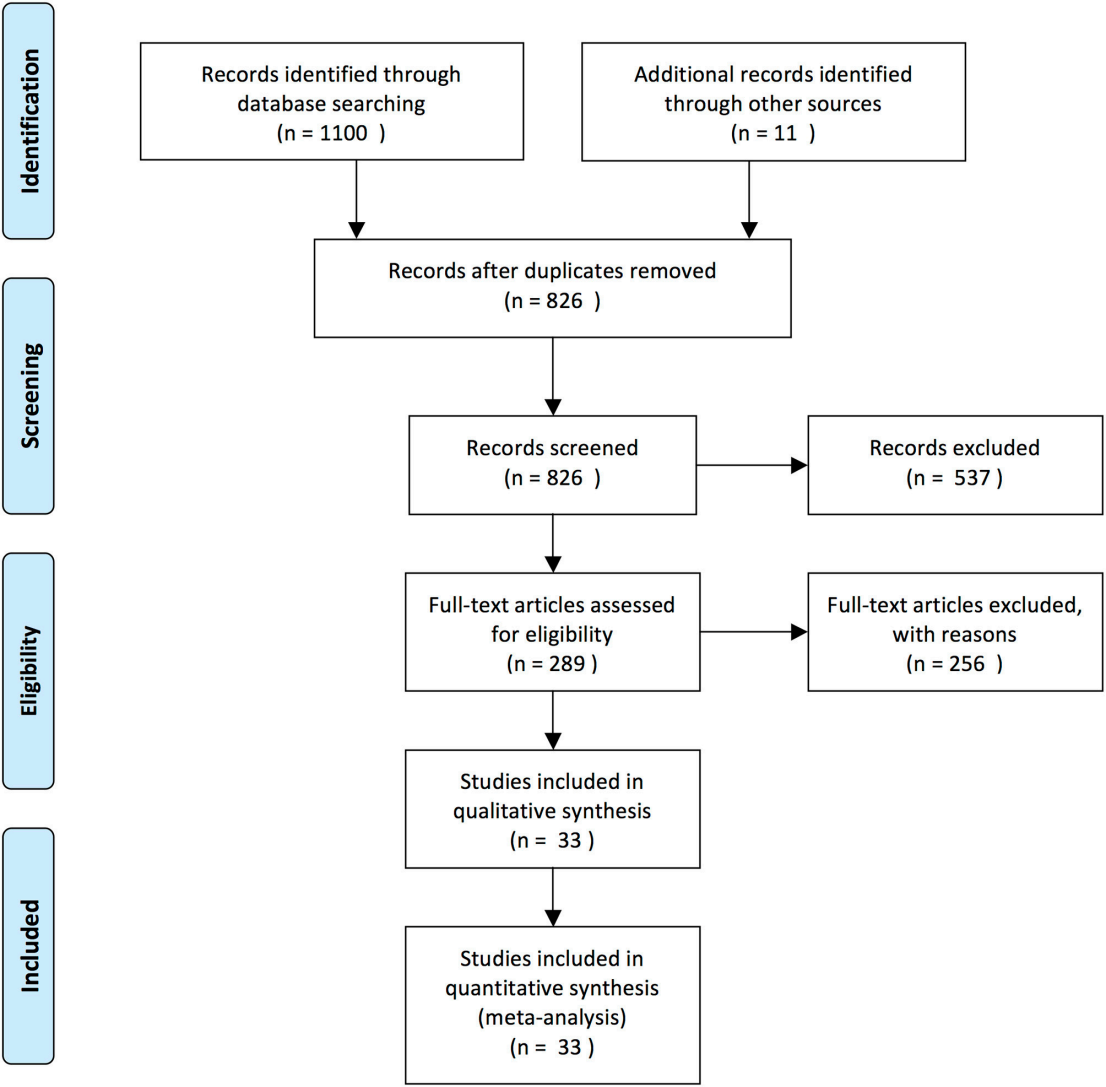
PRISMA flow diagram. The diagram for the study selection for this meta-analysis is based on the PRISMA-recommended flow chart (Moher et al., 2009).

Two independent authors read the articles for eligibility (age data from cohort and pilot studies) (A-ML, KM). The flow diagram recommended by the PRISMA guidelines shows the article selection procedure (Figure 2) (Moher et al., 2009). When conflicts arose, a third participant (PH) made the decision. Two authors collected data in an Excel file (Microsoft Corporation, Redmond, WA98052, USA) according to age (mean, median, range, standard deviation (SD) and interquartile range (IQR), where possible), study type, severity, mortality, and notes (A-ML, KM).

### Data Analysis

All meta-analytic calculations were performed with STATA software Version 11 (Stata Corporation, College Station, TX, USA). In our meta-analysis, the pooled effect sizes (ES) were the event rates with a 95% confidence interval (CI) for all outcomes. The random effect model by DerSimonian and Laird was used in all cases (DerSimonian and Laird, 1986). Heterogeneity was tested using Cochrane's Q and the I^2^ statistics. I^2^ statistics represent the percentage of effect size heterogeneity, which cannot be explained by random chance, but by other factors. *I*^2^-values of 25, 50, and 75% corresponded to low, moderate and high degrees of heterogeneity, based on the Cochrane handbook (Higgins, 2011). If the Q test is significant, it implies that the heterogeneity among effect sizes reported in the observed studies is greater than could be explained only by random error. We considered the Q test significant if *p* < 0.1. The forest plot was evaluated to represent the data. Publication bias was examined by visual inspection as asymmetry in the funnel plot and Egger's test (Sterne et al., 2001). A significant test result (*p* < 0.1) indicates the presence of bias.

A meta-regression was used to consider the effect of aging on mortality and severity. In both cases, we tested the hypothesis that all coefficients are zero. The results are provided as regression coefficients, 95% CIs, *p*-values and the explained variances of the models (R^2^ analogs).

A conventional regression analysis was also performed to confirm the results of the meta-regression. In this case, we used the pooled event rates from the subgroup analyses and the middle of the age subgroups as independent variables. We used the IBM SPSS Statistics software for these calculations (IBM Corporation, Armonk, New York, USA, Version 24).

### Quality Assessment

The quality of the articles was assessed by 3 main categories recommended by the modified Newcastle-Ottawa scale (Table 2, Supplementary Figure 1).

**Table 2:** The modified Newcastle–Ottawa quality assessment scale.

**Study**	**Sample size**	**Severe case**	**Mortality**	**Study type**	**Modified Newcastle-Ottawa quality assessment scale**									
					**Selection**				**Comparability**	**Outcome**				**Sum**
					**S1**	**S2**	**S3**	**S4**	**C1**	**O1.1**	**O1.2**	**O2**	**O3**	
Abou-Assi et al., 2002	156	5	14	Prospective	1	1	0	1	1	0	1	1	1	7
Albulushi et al., 2014	174	14	0	Retrospective	1	1	0	0	1	0	1	1	1	6
Gomez Beltran et al., 2013	24	1	0	Retrospective	1	1	1	1	1	0	1	1	1	8
de-Madaria et al., 2014	403	28	17	Prospective	1	1	1	1	0	1	0	1	1	7
Dombernowsky et al., 2016	359	nd	13	Retrospective	1	1	1	1	1	0	1	1	1	8
Gompertz et al., 2012	128	nd	2	Retrospective	1	1	1	1	0	0	0	1	1	6
Gompertz et al., 2013	1367	nd	115	Retrospective	1	1	1	1	0	1	0	1	1	7
Gonzalez-Gonzalez et al., 2012	605	nd	30	Prospective	1	1	1	1	1	1	1	1	1	9
Gornik et al., 2013	1058	210	41	Prospective	1	1	0	1	0	0	1	1	1	6
Gürleyik et al., 2005	55	13	1	Prospective	1	1	1	1	1	1	1	1	1	9
Karpavicius et al., 2016	102	20	5	Prospective	1	1	1	1	0	1	0	1	1	7
Knoepfli et al., 2007	310	63	8	Prospective	1	1	1	1	1	1	0	1	1	8
Lautz et al., 2011	211	nd	0	Retrospective	1	1	1	1	0	1	1	1	1	8
Milheiro et al., 1995	91	nd	10	Retrospective	1	1	0	1	0	0	0	1	1	5
Mole et al., 2016	2053	390	102	Retrospective	1	1	0	1	0	0	1	1	1	6
Muller et al., 2006	109	66	8	Prospective	1	1	1	1	1	1	1	1	1	9
Nijmeijer et al., 2013	622	119	20	Prospective	1	1	0	1	0	0	0	1	1	5
Ocampo et al., 2015	854	140	nd	Prospective	1	1	0	1	1	0	1	1	1	7
Pant et al., 2014	55012	nd	509	Retrospective	1	1	0	0	0	0	0	1	1	4
Parniczky et al., 2016	600	53	17	Prospective	1	1	1	1	1	1	1	1	1	9
Radenkovic et al., 2009	91	24	8	Prospective	1	1	1	1	0	1	0	1	1	7
Rashidi and Røkke, 2016	670	43	37	Prosp and Retrosp	1	1	1	1	1	1	0	1	1	8
Spanier et al., 2013	78257	nd	9515	Retrospective	1	1	0	1	1	0	1	1	1	7
Uomo et al., 2007	1173	167	36	Prospective	1	1	1	1	0	1	1	1	1	8
De Waele et al., 2007	40	14	6	Retrospective	1	1	1	1	0	1	0	1	1	7
Wang et al., 2015	120	31	13	Retrospective	1	1	0	1	0	0	0	1	1	5
Ho et al., 2015	12284	765	nd	Retrospective	0	1	0	1	0	0	0	1	1	4
Weitz et al., 2016	346	21	12	Retrospective	1	1	1	1	0	1	1	1	1	8
Wu et al., 2008	36178	nd	569	Retrospective	1	1	0	0	0	0	0	1	1	4
Yeung et al., 1996	43	nd	1	Retrospective	1	1	0	0	1	0	1	1	1	6
Yue et al., 2015	169	68	nd	Prospective	1	1	0	1	0	0	0	1	1	5
Zhang et al., 2016	974	223	58	Retrospective	1	1	1	1	0	1	1	1	1	8
Zuidema et al., 2014	64	11	3	Prospective	1	1	0	1	0	0	0	1	1	5

*Ranks in three categories (green-1: low risk; red-0: high risk, yellow-0: unclear risk) are shown. S1, non-selected etiology AP; S2, all participants have an AP diagnosis; S3, AP diagnosis is confirmed using the latest guidelines; S4, non-selected severity cases. C1: comparability defined by exact age ranges in years. O1.1, severity assigned according to the latest guidelines; O1.2, described mortality (in-hospital and pancreas-related); O2–O3, adequate follow-up for outcome occurrence morality and severity*.

## Results

### Flow Diagram of Studies Retrieved for the Review, Study Selection, and Characteristics

Our search yielded 1,100 articles (704, 379, and 17 in Embase, PubMed, and Cochrane, respectively) (Figure 2). Eleven additional articles were found with potential data eligibility for the meta-analysis in the references of the primarily selected articles. After excluding duplicates and irrelevant articles, a total of 33 articles involving 194,702 patients met the inclusion criteria (Table 2).

### Synthetized Findings

#### Severity

A total of 23 studies with 22,451 patients were suitable for analyzing severity (Tables 2, 3) (Abou-Assi et al., 2002; Gürleyik et al., 2005; Muller et al., 2006; De Waele et al., 2007; Knoepfli et al., 2007; Uomo et al., 2007; Radenkovic et al., 2009; Gomez Beltran et al., 2013; Gornik et al., 2013; Nijmeijer et al., 2013; Albulushi et al., 2014; de-Madaria et al., 2014; Zuidema et al., 2014; Ho et al., 2015; Ocampo et al., 2015; Wang et al., 2015; Yue et al., 2015; Karpavicius et al., 2016; Mole et al., 2016; Parniczky et al., 2016; Rashidi and Røkke, 2016; Weitz et al., 2016; Zhang et al., 2016). Two thousand Four Hundred Eighty Nine severe cases were found divided into seven age groups with a low severity rate under 30 years. There was a low incidence severe AP rate in patients under 30 and rose continuously between ages 30 and 70 (Table 3).

**Table 3:** Data of patient's number and severe cases in age groups.

**Age**	**Severe AP**	**Patient no**.	**%**
U20	1	24	4.2
20–29	0	36	0.0
30–39	5	75	6.7
40–49	726	7882	9.2
50–59	1352	11933	11.3
60–69	390	2344	16.6
A70	15	157	9.6
Sum	2489	22451	11.1

*There was only one severe AP in patients under 30; however, the incidence of severe AP rose continuously between ages 30 and 70*.

**Figure 3:**
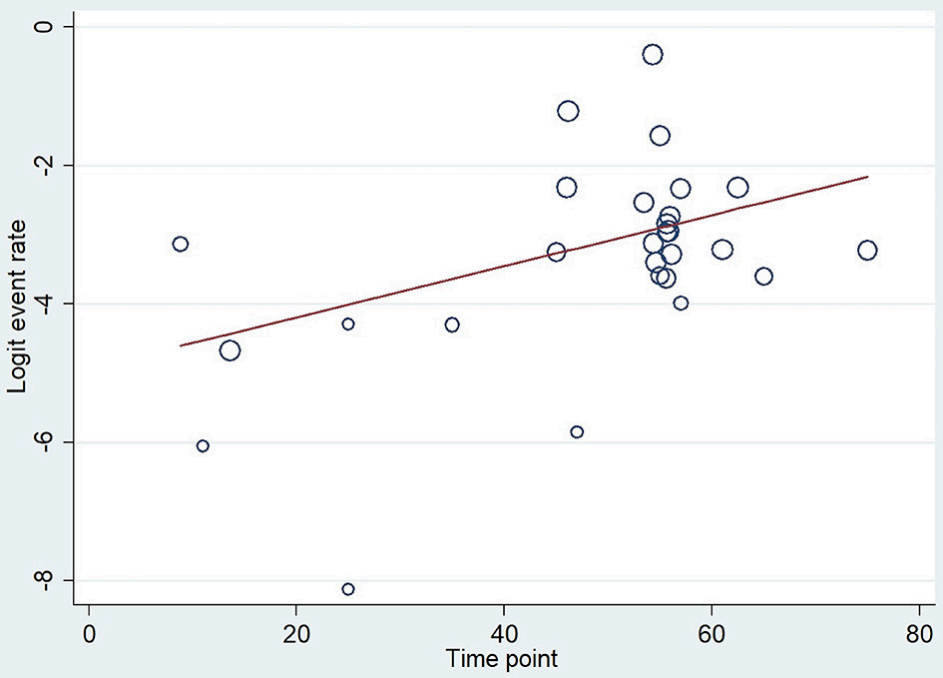
Meta-regression of severity. The figure shows 29 data from 23 reports where x = age (mean), y = logit event rate: ln[p/(1-p)], and circle diameters show the weight of each study based on the random effect model. The meta-regression shows a significant (*p* < 0.001) relationship between age and severity (*r*^2^ = 31.6), therefore the risk for developing severe cases is elevated by aging.

**Figure 4:**
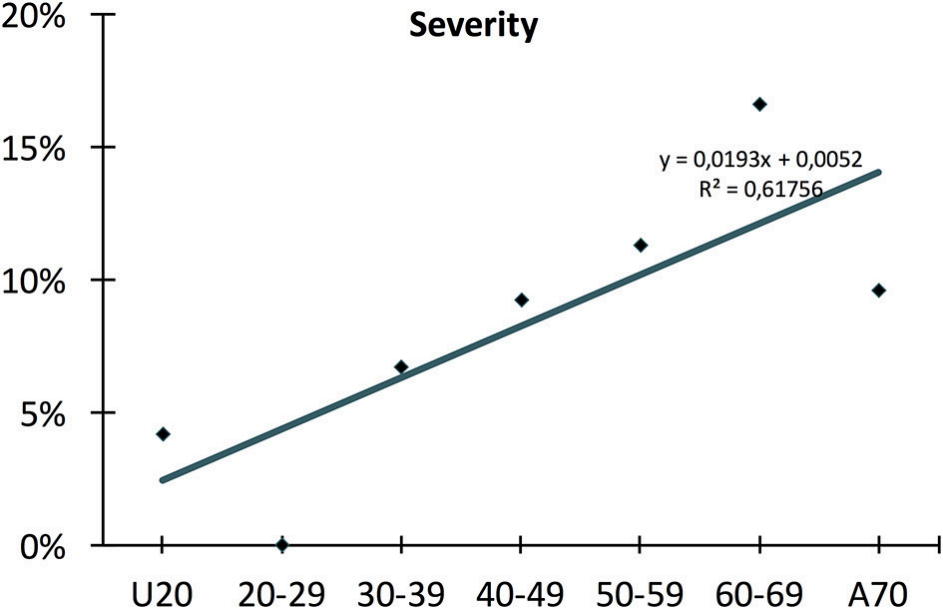
Conventional regression of severity. The conventional regression, which is independent of distortion from diverse numbers of patients, shows a linear rise (0.193%/year) in severity from young to old age.

Firstly, a meta-regression was performed to investigate the relationship between age and severity (Figure 3). The number of patients in each age group category was extremely diverse (between 24 and 11,933); however, a significant relationship was detected (coefficient: 0.035 CI: 0.019–0.052, *p* < 0.001; adjusted r^2^: 31.6%). A conventional regression analysis was also performed showing a linear increase (0.193%/year) from ages U20 to A70 (Figure 4).

**Figure 5:**
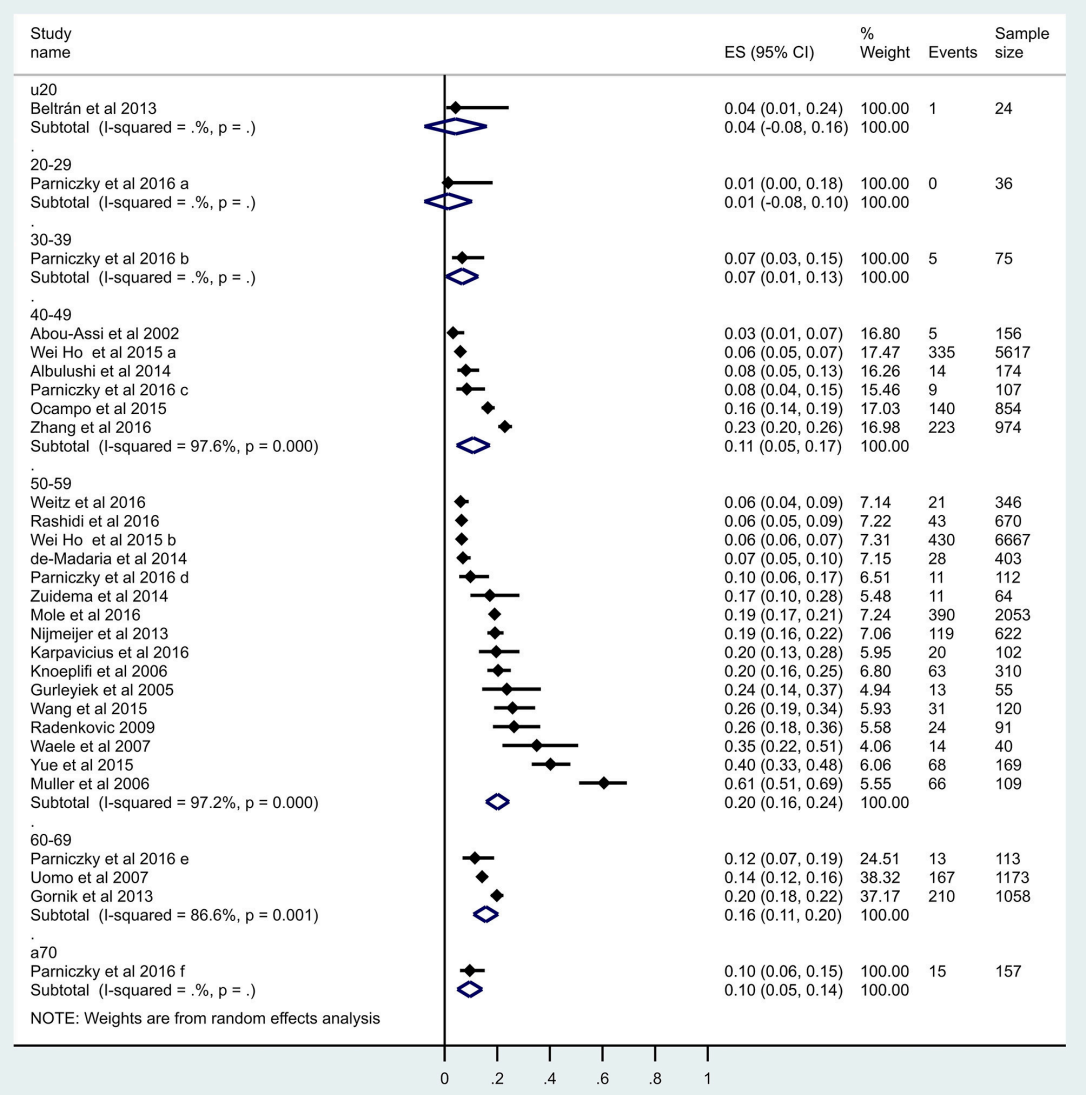
Forest plot of studies evaluating severity in acute pancreatitis in age groups. Full diamonds show the weighted event rates for studies, respectively, line represents the 95% confidence interval (CI), and empty diamonds show the pooled results of severe cases with a steadily rising frequency from young to older age. Wideness of the empty diamond represents the confidence limits. Under 40 there is a slight elevation concerning severe cases, from 40 to 60 severity rates differs in the studies, then A60 remains stable.

This continuous elevation was also confirmed by forest plot (Figure 5). There was 1 severe AP U20: 4.2% (1/24; pooled event rate: 0.042 CI: −0.077–0.161); 20–29: 0% (0/36; pooled event rate: 0.014 CI: 0.077–0.104); 30–39: 6.7% (5/75; pooled event rate: 0.067 CI: −0.005–0.128); 40–49: 9.2% (726/7882; pooled event rate: 0.109 CI: 0.046–0.172); 50–59: 11.3% (1352/11 933; pooled event rate: 0.201 CI: 0.158–0.245); 60–69: 16.6% (390/2344; pooled event rate: 0.157 CI: 0.110–0.203); A70: 9.6% (15/157; pooled event rate: 0.096 CI: 0.049–0.143). In sum, 11.1% (2489/22 451).

Publication bias was tested by inspection of funnel plot and Egger's test (CI: 1.961–6.728; *p* = 0.001). The visible asymmetry (plots are mostly concentrated to the right side) is most probably due to the fact that authors mostly present data with high volume examinations (Supplementary Figure 2).

**Figure 6:**
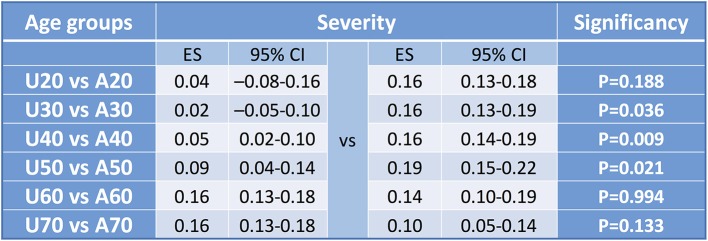
Forest plot results for cut-off values for severity. Summary table of pooled effect with CI and significance levels to detect cut off value. Concerning mortality all comparisons were significant, however examining severity only three. Explanation might be that in young ages there is a low event rate, in middle age groups there is a higher proportion therefore the difference is equalized leading to a non-significant difference. The same occur in the aged vs. middle aged groups.

The cut-off values in sorting articles to U20 and A20, U30 and A30, U40 and A40, U50 and A50, U60 and A60, and U70 and A70 (Supplementary Figures 3–8) resulted in significant differences considering three comparison, respectively (U30 vs. A30 *p* = 0.036; U40 vs. A40 *p* = 0.009; U50 vs. A50 *p* = 0.021) (Figure 6).

In addition, we performed several sub-group analysis in order to decrease the heterogeneity in our study. Firstly, we used articles only where severity was assessed by the Atlanta or the revised Atlanta classification. This additional analysis could largely decrease the heterogeneity [I^2^ = 40–49: 0%, 50–59:96.9%, 60–69:86.6% (Supplementary Figure 9)]. Secondly, we excluded the low quality (NOS 4 and 5) studies from the analysis. This analysis also could improve the heterogenity [(I^2^ = 40–49: 96.3%, 50–59:96.5%, 60–69:86.6% (Supplementary Figure 10)].

And finally, we excluded studies from the analysis where age ranges might overlap between the groups because of given age ranges. We could also successfully decrease the heterogeneity [(*I*^2^ = 40–49: 98%, 50–59:97.1%, 60–69:86.6% (Supplementary Figure 11)].

Importantly, none of them modified the outcome of the study which decrease the overall limitations of our results.

#### Mortality

**Figure 7:**
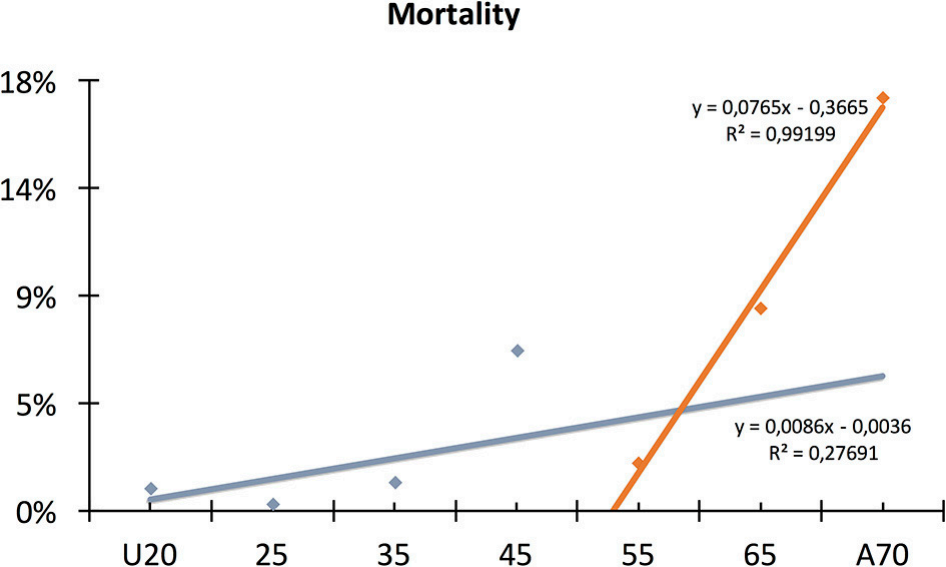
Conventional regression of mortality. The conventional regression shows a linear elevation until 59, however from this age the mortality rate started elevating with 9 times higher rate until the age of 70.

**Figure 8:**
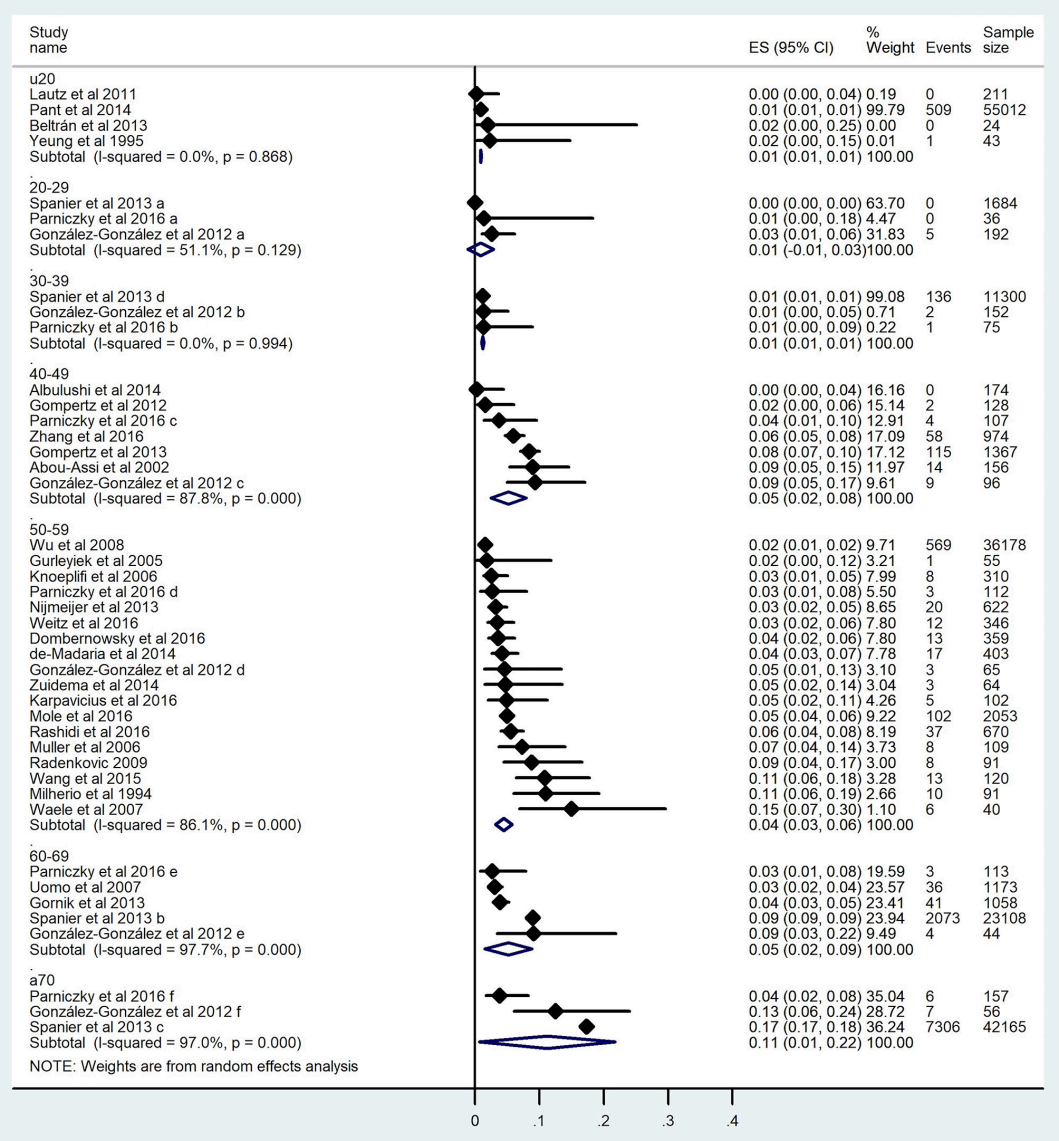
Forest plot of studies evaluating mortality in acute pancreatitis. Full diamonds show the weighted event rates for studies, respectively, line represents the 95% confidence interval (CI), and empty diamonds show the pooled results of mortality with a steadily rising frequency from young to older age. Wideness of the empty diamond represents the confidence limits. The diamonds show a steadily rising frequency in mortality from youth to old age.

Thirty studies involving 181,395 subjects contained data on mortality (Milheiro et al., 1995; Yeung et al., 1996; Abou-Assi et al., 2002; Gürleyik et al., 2005; Muller et al., 2006; De Waele et al., 2007; Knoepfli et al., 2007; Uomo et al., 2007; Wu et al., 2008; Radenkovic et al., 2009; Lautz et al., 2011; Gompertz et al., 2012, 2013; Gonzalez-Gonzalez et al., 2012; Gomez Beltran et al., 2013; Gornik et al., 2013; Nijmeijer et al., 2013; Spanier et al., 2013; Albulushi et al., 2014; de-Madaria et al., 2014; Pant et al., 2014; Zuidema et al., 2014; Wang et al., 2015; Dombernowsky et al., 2016; Karpavicius et al., 2016; Mole et al., 2016; Parniczky et al., 2016; Rashidi and Røkke, 2016; Weitz et al., 2016; Zhang et al., 2016) (Tables 2, 4). Eleven thousand one hundred and seventy deceased cases were found in the seven age groups with the highest rates in groups 40–49 and A60 (Table 4). Considering that a severe course of AP increases the risk for mortality, we expected a similar regression to severity (Figure 4). The mortality rate was 0.9% in patients under 20 and demonstrated a continuous, linear elevation until 59, however from this age the mortality rate started elevating with 9 times higher rate until the age of 70 (Figure 7). The mortality rate grew 0.086%/year between ages 20 and 59 and 0.765%/year between 59 and 70 (Figure 7). Overall, patients above 70 had a mortality rate 19 times higher than those under 20 (Table 4). The mortality rate rising with age was also confirmed by forest plot, showing a clear elevation from pediatric to elderly patients: U20: 0.9% (510/55 290; pooled event rate: 0.009 CI: 0.008–0.010); 20–29: 2.6% (5/1912; pooled event rate: 0.009 CI: −0.011–0.029); 30–39: 1.2% (139/11 527; pooled event rate: 0.012 CI: 0.010–0.014); 40–49: 6.7% (202/3002; pooled event rate: 0.052 CI: 0.025–0.079); 50–59: 2% (838/41 634; pooled event rate: 0.045 CI: 0.032–0.057); 60–69: 8.5% (2153/25 452; pooled event rate: 0.052 CI: 0.015–0.088); and A70: 17.3% (7312/42 322; pooled event rate: 0.112 CI: 0.007–0.217) (Figure 8). In summary, 6.2% (11 170/181 395).

**Table 4:** Data of patient's number and deceased cases in age groups.

**Age**	**Fatal event**	**Patient no**.	**%**
U20	510	55290	0.9
20–29	5	1912	0.26
30–39	139	11527	1.2
40–49	202	3002	6.7
50–59	838	41790	2.0
60–69	2157	25496	8.5
A70	7319	42378	17.3
Sum	11170	181395	6.2

*The incidence of severe AP rose continuously between ages 30 and 70*.

**Figure 9:**
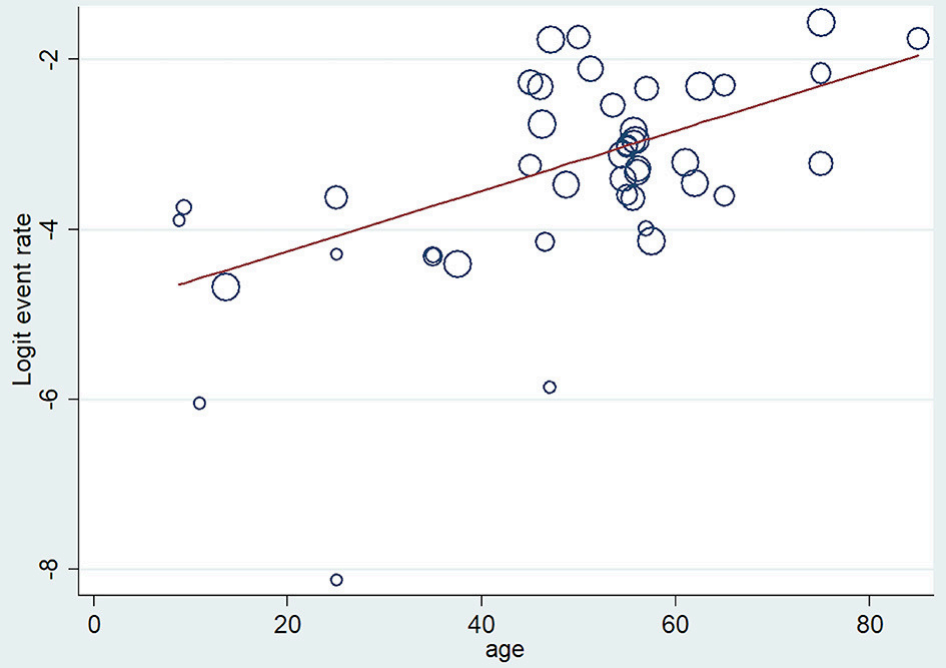
Meta-regression of mortality. The figure shows 43 data from 30 reports where x = age (mean), y = logit event rate: ln[p/(1-p)], and circle diameters show the random size of each study. The meta-regression shows a significant relationship (*p* = 0.022) between age and mortality.

A meta-regression analysis on mortality showed a significant difference (coefficient: 0.037 CI: 0.006–0.068, *p* = 0.022; adjusted *r*^2^: 13.8%, Figure 9). Publication bias was tested by funnel plot and Egger's test (CI: −0.901–9.234; *p* = 0.104) and showed mild asymmetry, but based on Egger's test publication bias was unlikely (Supplementary Figure 12).

**Figure 10:**
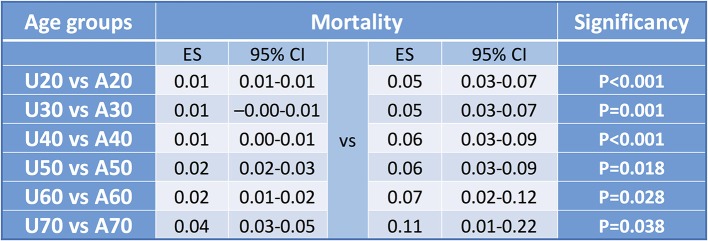
Forest plot results for cut-off values for mortality. Forest plot results from studies evaluating the cut-off values for mortality in acute pancreatitis with significant results in each of four groups. All comparisons showed a significant difference.

Forest plot analyses comparing U20 to A20, U30 to A30, U40 to A40 and U50 vs. A50 showed significant differences, respectively (U20 vs. A20 *p* < 0.001; U30 vs. A30 *p* = 0.001; U40 vs. A40 *p* < 0.001; U50 vs. A50 *p* = 0.018; U60 vs. A60 *p* = 0.028, and U70 vs. A70 *p* = 0.038) (Supplementary Figures 13–18). Forest plot results are summarized in Figure 10.

We excluded the low quality (NOS 4 and 5) studies from the analysis to lower the heterogeneity [*I*^2^ = 40–49: 96.3%, 50–59:96.5%, 60–69:86.6% (Supplementary Figure 19)].

### Risk of Bias and Quality Assessment

The risk of bias was examined by funnel plot and Egger's test (see above severity and mortality). The quality of the included articles were assessed by using the modified Newcastle–Ottawa scale as described earlier (Deeks et al., 2003; Mata et al., 2015; Rotenstein et al., 2016).

Two independent investigators have evaluated the articles and classified using a clear guidance described in Supplementary Figure 1. The following three main categories were applied: (i) selection of study groups (including four subgroups: S1: non-selected etiology AP; S2: all participants have an AP diagnosis; S3: AP diagnosis is confirmed using the latest guidelines; S4: non-selected severity cases); (ii) comparability of the groups (C1: comparability defined by exact age ranges in years); and (iii) outcome of interest (including four subgroups: O1.1: severity assigned by the latest guidelines; O1.2 described mortality (in-hospital and pancreas-related); and O2–O3: adequate follow-up for outcome occurrence, morality and severity). Each item was marked: green-1: low risk; red-0: high risk and yellow-0: unclear risk of bias. A total of 9 points was the maximum that could be assigned (Table 2) (Milheiro et al., 1995; Yeung et al., 1996; Abou-Assi et al., 2002; Gürleyik et al., 2005; Muller et al., 2006; De Waele et al., 2007; Knoepfli et al., 2007; Uomo et al., 2007; Wu et al., 2008; Radenkovic et al., 2009; Lautz et al., 2011; Gompertz et al., 2012, 2013; Gonzalez-Gonzalez et al., 2012; Gomez Beltran et al., 2013; Gornik et al., 2013; Nijmeijer et al., 2013; Spanier et al., 2013; Albulushi et al., 2014; de-Madaria et al., 2014; Pant et al., 2014; Zuidema et al., 2014; Ho et al., 2015; Ocampo et al., 2015; Wang et al., 2015; Yue et al., 2015; Dombernowsky et al., 2016; Karpavicius et al., 2016; Mole et al., 2016; Parniczky et al., 2016; Rashidi and Røkke, 2016; Weitz et al., 2016; Zhang et al., 2016).

Whenever different points were given by the investigators a third member of the team made the final decision.

## Discussion

### Summary of Main Findings

Here we provide the first detailed meta-analysis on the effects of aging on AP. Aging has been demonstrated to play an important role in AP; however, due to the lack of detailed mathematical analysis, there is a great difference between the cut-off values used in predictive scoring systems (Blamey et al., 1984; Wagner and Draper, 1984; Legall et al., 1993; Hirota et al., 2006; Spitzer et al., 2006; Wu et al., 2008).

With regard to severity, unfortunately we only have two articles in which severity was one of the outcome parameters in youth. In one of these studies, Párniczky et al. found no severe cases in the 36 patients under 30 years of age (Parniczky et al., 2016). Similarly, Beltrán et al. found only a single severe case in cohort of 24 patients suggesting a low incidence rate of severe AP in youth (Gomez Beltran et al., 2013). Our situation was far easier regards mortality as data from large nationwide cohorts were available. In a large epidemiology study involving 55,012 patients under 20 years in the USA, Pant et al. showed that mortality is only 0.92% (Pant et al., 2014). Others have also described low mortality in smaller cohorts. Lautz et al. found 0% (0/211 patients) mortality under 20 years, while Yeung et al. reported 2.33% (1/43 patients) (Yeung et al., 1996; Lautz et al., 2011). In contrast, no mortality was found among 1,720 patients between the ages of 20 and 29 in a Hungarian and a Dutch cohort (Spanier et al., 2013; Parniczky et al., 2016). Middle-aged patients (30–59 y) had a mortality rate more than two times higher (Abou-Assi et al., 2002; Gürleyik et al., 2005; Muller et al., 2006; De Waele et al., 2007; Knoepfli et al., 2007; Wu et al., 2008; Radenkovic et al., 2009; Nijmeijer et al., 2013; Spanier et al., 2013; Albulushi et al., 2014; de-Madaria et al., 2014; Zuidema et al., 2014; Wang et al., 2015; Dombernowsky et al., 2016; Karpavicius et al., 2016; Mole et al., 2016; Parniczky et al., 2016; Rashidi and Røkke, 2016; Weitz et al., 2016; Zhang et al., 2016).

Our second main observation was that up until 59 years (this cut-off value was mathematically calculated), both severity and mortality rise linearly (Figures 4, 7). The rate of severity increases 0.193%/year, and mortality grows 0.086%/year. It has been documented that almost all death cases come from the severe AP group; therefore, we can assume that although the number of severe cases rises every year, the risk for mortality in severe AP remains constant at around 20% (Parniczky et al., 2016).

Thirdly, we found that above 59 years the mortality rate rapidly increases; meanwhile, the rate of severe pancreatitis follows the earlier, slightly elevated pattern (Figures 4, 7). These data clearly suggest that additional factors which are lacking or rare below 59 years also affect mortality in AP. One of the best candidates responsible for the increased elevation of mortality in elderly is definitely co-morbidity. It has been shown that the burden of co-morbidities increases with age (Vasilopoulos et al., 2014; Murata et al., 2015). In addition, it has been also reported that the outcome of AP is worsen by severe co-morbidities (Frey et al., 2007; Murata et al., 2011). Therefore, we can hypothesize that the elevation of severity and mortality with age is attributed to co-morbidity rather than aging.

The incidence of severe AP in patients, however, showed a continuous, linear rise between the ages of 20 and 70 (0.193%/year) of up to 16.6%. The mortality rate was 0.9% in patients under 20 and demonstrated a continuous increase until the age of 70. The mortality rate between 20 and 59 grew 0.086%/year and 0.765%/year between 59 and 70. Overall, patients above 70 had a mortality rate 19 times higher than patients under 20. The rise of mortality rate with age was thus also confirmed.

In adults, the severity of AP clearly increases with age. With regard to mortality, it follows a similar linear rise until 59 years; however, after that a 9-fold change is observed in its steepness. This result completely confirms the observation of Ranson et al. that age is associated with a significantly increased risk of death over 55 years (Ranson and Pasternack, 1977; Blamey et al., 1984). Imrie et al. (1978) modified the scoring system; however, they still considered age above 60 as a valuable parameter. Blamey et al. (1984) evaluated a prospective study with 347 patients in a seven-year period to simplify the system and to improve its accuracy. With regard to age, they also found the cut-off point at 55 years.

The BISAP scoring system was established as the first population-based prognostic scoring system in order to evaluate the risk of in-hospital mortality prior to the onset of organ failure (Wu et al., 2008). The CART analysis identified age above 60 years for prediction of in-hospital mortality based on parameters collected in 2000–2001 in the first 24 h from a patient population of 17,922 suffering from AP (Wu et al., 2008).

**Figure 11:**
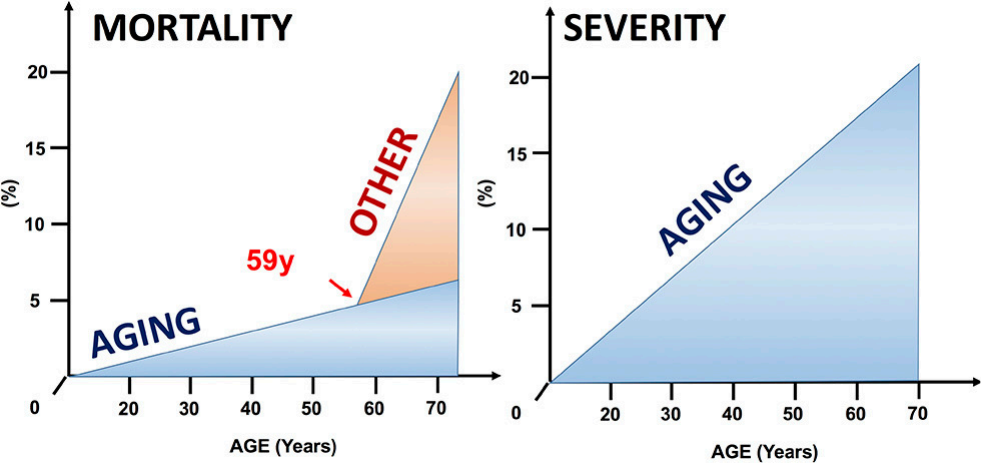
Factors that may prepossess mortality and severity in AP. Our data show that age linearly correlates to higher risk of developing severe AP. Concerning mortality other factors may elevate the risk of decease cases above 59 years of age.

In summary, the predictive scoring systems correspond with our results, which suggests that mortality rises quickly above 59 years of age. Our data suggest that other factors which are associated with older age elevate the mortality in AP (Figure 11).

One of the candidates is definitely comorbidity. Fan et al. in 1988 also raised the question and found that concomitant medical and surgical diseases were responsible for the higher in-hospital mortality rate in elderly rather that consequences of AP (Fan et al., 1988). However, they also observed a higher incidence of not local, but systemic complications in older age. They concluded that, if concomitant diseases were ignored, the difference in mortality rate between young and elderly disappeared (Fan et al., 1988). Charlson et al. (1994) validated an Age-Adjusted Charlson comorbidity index (CCI) showing the absent of age from CCI index. Forty years of age have the lowest risk of comorbid death, moreover each decade of age over 40 adds 1 extra point to the risk which is added to the calculated CCI score.

A currently revealed propensity score-matched analysis examined the mortality and severity in the elderly in ABP (Patel et al., 2018). They grouped 184,763 patients in two age groups (< 65 years of age vs. ≥65 years) and found that the index admission mortality rate for the elderly was significantly higher (0.32% (*n* = 356) vs. 1.96% (*n* = 1473); *p* < 0.001). The odds of mortality increased progressively in patients aged 75 to 84 years (OR 1.39; 95% CI: 1.06–1.82) and 85 years or older (OR 2.21; 95% CI: 1.70, 2.86). Further, increasing age was also associated with higher odds of severe AP (75 to 84 years: OR 1.20; 95% CI: 1.12, 1.30; 85 y or older: OR 1.28; 95% CI: 1.17, 1.40). However, elderly patients in this analysis had significantly higher ≥3 co-morbidities (based on an Elixhauser score of < 3 and ≥ 3) (OR 4.59; 95% CI: 4.33, 4.87; *p* < 0.001), they concluded that age independently contributes to increased mortality in ABP.

However, in order to prove the influence of comorbidity on survivals, we wanted to extend our study with comparing comorbidities at different age categories. Since the articles in this study did not contain sufficient amount of information on comorbidities we have performed a large multinational cohort analysis on a prospective high quality database (Szakács et al., 2018). The analysis of a total of 1,203 patients showed that severe comorbidities (CCI≥3) predict mortality (OR = 4.48; CI: 1.57–12.80) much better than age, suggesting that comorbidity is an important additional predictor for mortality. More details of this investigation can be found in the forthcoming article in Frontiers Physiological Sciences entitled: “Aging and comorbidities in acute pancreatitis II: A cohort-analysis based on 1 203 prospectively collected cases from 12 countries” (DOI: 10.3389/fphys.2018.01776).

### Strengths and Limitations

Strength 1 This systematic review and meta-analysis is based on a database which is at least 10 times greater in volume than the database used to develop the largest scoring system
  Strength 2 Patients were included independently of etiologies, nationalities, severities and ages, without any limitations in this study.
  Strength 3 Aging has serious impact on the healthcare systems worldwide; therefore, scientists' attention must focus on geriatrics.
  Limitation 1 In most of the articles, the age of the patients was published in median, mean or IQR; therefore, distortion was alerted.
  Limitation 2 The severity scoring guidelines have changed considerably over the years; therefore, there might be cases in which severities have been misclassified in the studies under analysis compared to our current knowledge.
  Limitation 3 The co-morbidities of patients involved in the analysis are unknown; therefore, the decisive question as to whether age or age-associated co-morbidity plays an aggravating role remains unanswered in this meta-analysis.
  Limitation 4 The large variety of studies caused high heterogeneity which may indicate hidden distorting factors in this analysis.
  Limitation 5 We could not explain the reason why the mortality of the 50–59-year age group is lower than that of the 40–49-year age group. Therefore, it cannot exclude the possibility that the mortality rate is monophasic and the cut off A70 is better than the cut off of 59.

## Conclusions

In conclusion, our analysis shows that age has an effect on AP. Both severity and mortality rise linearly, however the rate of elevation in mortality is 9 times higher above 59 than below. Our results rise an important question whether a restorative role is played by aging or other factors like co-morbidity.

## Core Tip

There has been a dramatic increase in life expectancy over the last few centuries. In addition, the incidence rate of one of the most common gastrointestinal disorders, acute pancreatitis (AP), is also growing. Here we provide a detailed mathematical analysis of the effects of aging on AP. Our data clearly shows that (1) younger age has a protective effect in AP, (2) aging raises both the severity and mortality of AP, and, importantly, (3) the mortality rate for patients above 59 years rises with 9 times greater intensity than that in younger patients.

## Author's Note

The results of this article suggested clearly that additional factors play a crucial role in mortality above 59 years of age (Figures 7, 11). There is a Part II of this publication in which a detailed analysis of a 1,203 prospectively collected cases showed that comorbidity is the key factor (Figure 5 - https://www.frontiersin.org/articles/10.3389/fphys.2018.01776/full; doi: 10.3389/fphys.2018.01776).

## Author Contributions

KM and A-ML conducted the database search and read the articles for eligibility; when a conflict arose, a third participant, PH, made the decision. KM and A-ML collected the data from the articles in an Excel file. NF and PM analyzed the data. PS, ZR, and LC performed the bias analysis and quality assessment. KM, TH, and BE drafted the manuscript. ÀV, GV, TH, A-ML, MO, LC, PS, ZR, IC, and PH edited the manuscript. KM, MO, and A-ML edited the tables and figures. PS and ZR completed the PRISMA checklist. PH made the critical revision on the finalized manuscript. All authors have read and approved the final manuscript.

### Conflict of Interest Statement

The authors declare that the research was conducted in the absence of any commercial or financial relationships that could be construed as a potential conflict of interest.

## Abbreviations

A70: above 70 years
ABP: acute biliary pancreatitis
AP: acute pancreatitis
APACHE II: Acute Physiology and Chronic Health Evaluation
BALI: BUN, Age, LDH, IL-6
BISAP: Bedside Index for Severity in Acute Pancreatitis score
CI: confidence interval
ES: effect sizes
IQR: interquartile range
JNP: Japanese Severity Score
OR: odd's ratio
U20: under 20 years
PRISMA: preferred reporting items for systematic review and meta-analysis statement
SAPS II: Simplified Acute Physiology Score
SD: standard deviation.
