# Supplementary material for: Aging and Comorbidities in Acute Pancreatitis I: A Meta-Analysis and Systematic Review Based on 194,702 Patients
Source: Front Physiol. 2019 Apr 2;10:328. doi: 10.3389/fphys.2019.00328 (PMC6454469; doi:10.3389/fphys.2019.00328)
Supplement: Supplementary file 1 [file Data_Sheet_1.pdf]

Assessment of the modified Newcastle-Ottawa scale in our study				
	(i) selection of study groups			
	S1	S2	S3	S4
**low risk**	non-selected etiology	confirmed AP diagnosis	AP diagnosis is confirmed using the latest guidelines	non-selected severity cases
**high risk**	selected etiology	unstable diagnosis concering the IAP/APA guideline	AP diagnosis is made using not the latest guidelines	selected severity cases
**unclear risk**	not mentioned	not mentioned	no information about diagnosis critera	no information
	(ii) comparability of the groups			
	C1			
**low risk**	exact age range is provided			
**unlcear risk**	age range is not provided			
	(iii) outcome of interest			
	O1.1	O1.2	O2	O3
**low risk**	severity assigned by the latest guidelines	described mortality (in-hospital and pancreas-related)	adequate follow-up for outcome occurrence (morality)	adequate follow-up for outcome occurrence (severity)
**high risk**	severity assigned by not the latest guidelines	in-hospital and pancreas-related	not adequate follow-up for outcome occurrence (morality)	not adequate follow-up for outcome occurrence (morality)
**unclear risk**	not mentioned	not described mortality	no information about follow-up	no information about follow-up

Suppl. Fig. 1. Assessment of the modified Newcastle-Ottawa scale. Low risk equals one point, low and unclear risk equals no point (see Table 2).

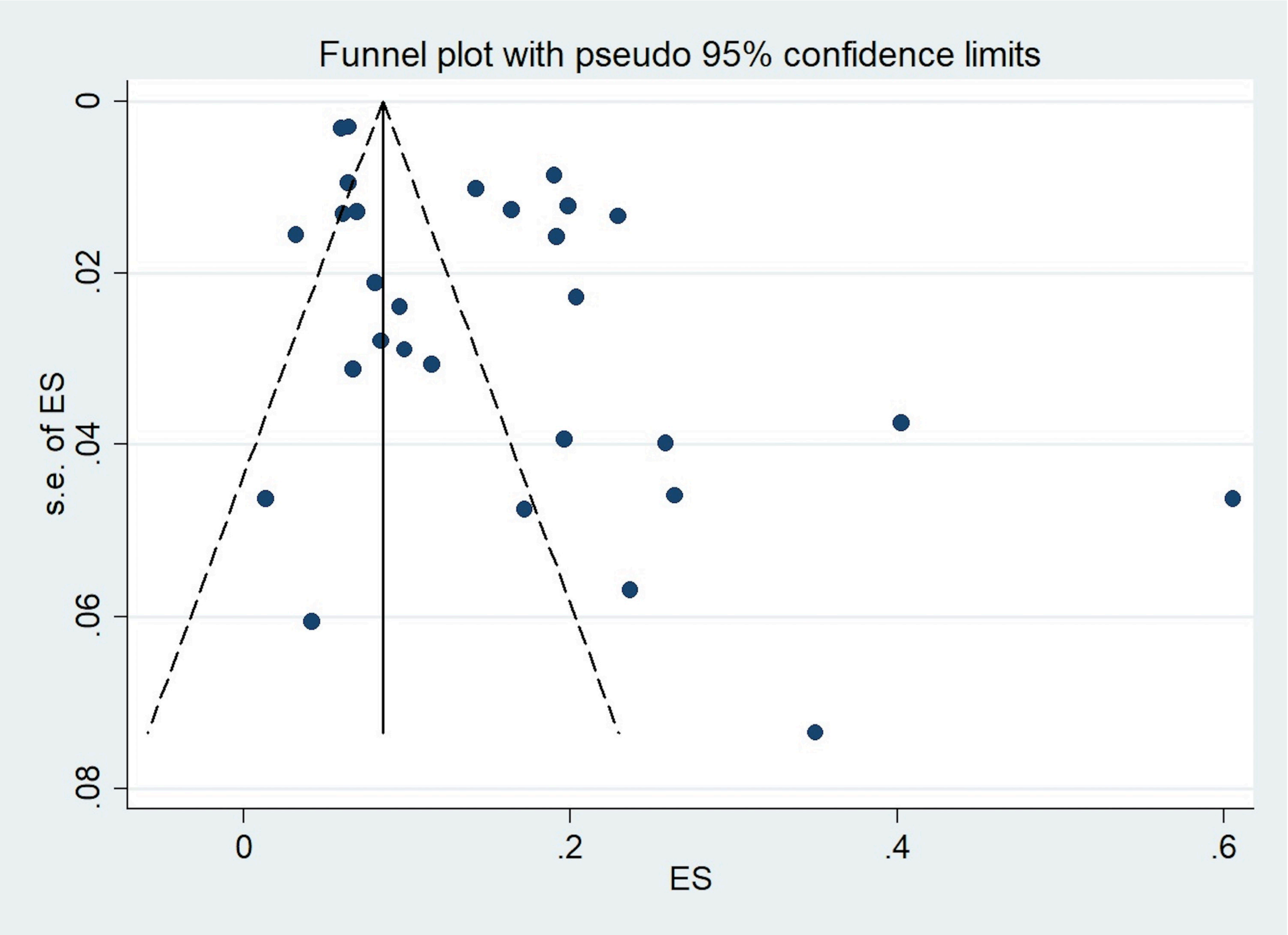

Suppl. Fig. 2. Funnel plot of severity in terms of publication bias. Funnel plots represent the standard error (SE) plotted against event rates (ES) for each study. The dotted line shows the 95% confidence limits. Plots are mostly on the right side showing that publication bias might present (CI: 1.961–6.728; p=0.001).

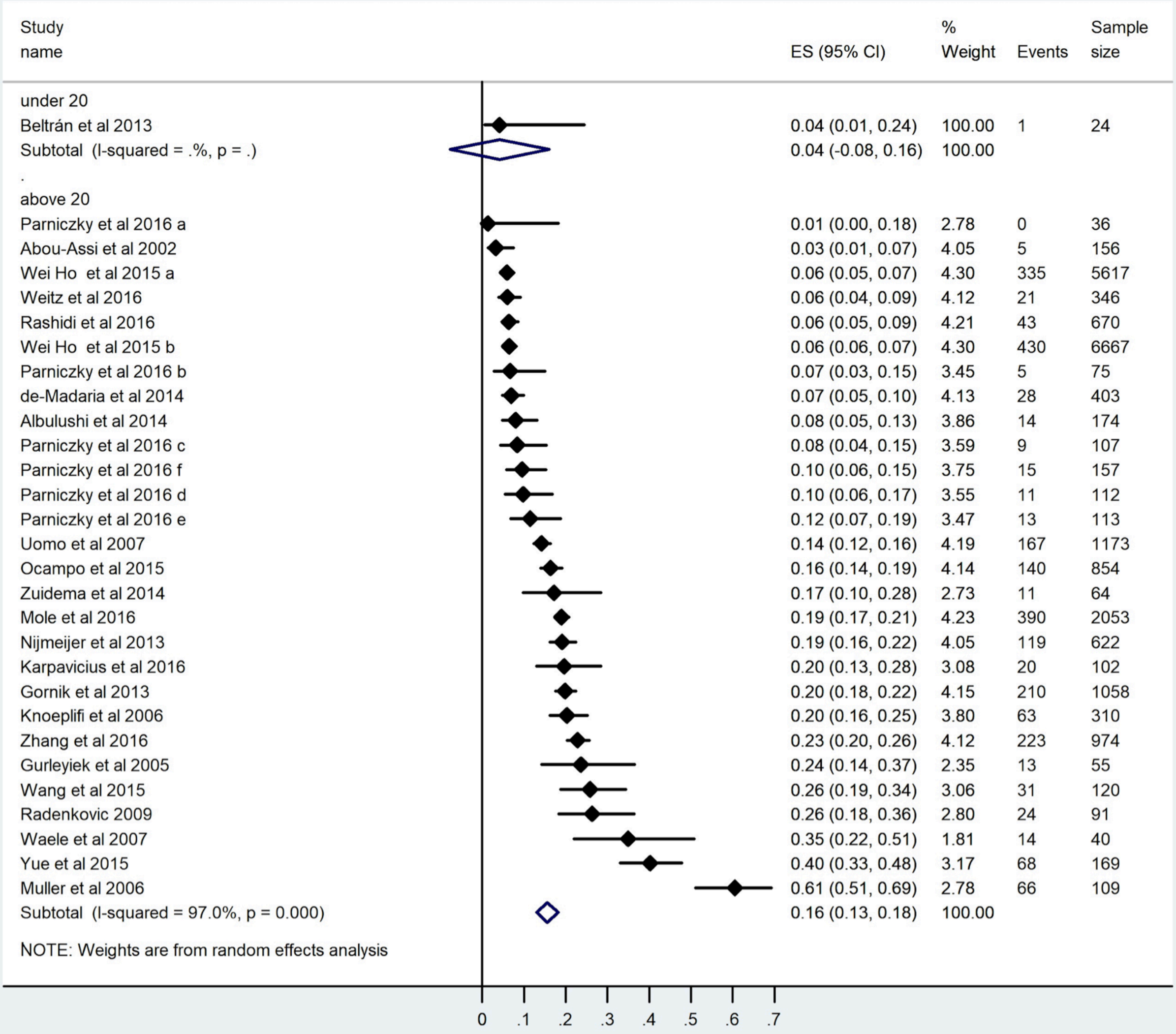

Suppl. Fig. 3. Forest plot of studies evaluating severity at age U20 compared to A20. Full diamonds show the weighted event rates for studies respectively, line represents the 95% confidence interval (CI), and empty diamonds show the pooled results of severe cases. A non-significant difference can be observed in severity under 20 and above 20 (p=0.188).

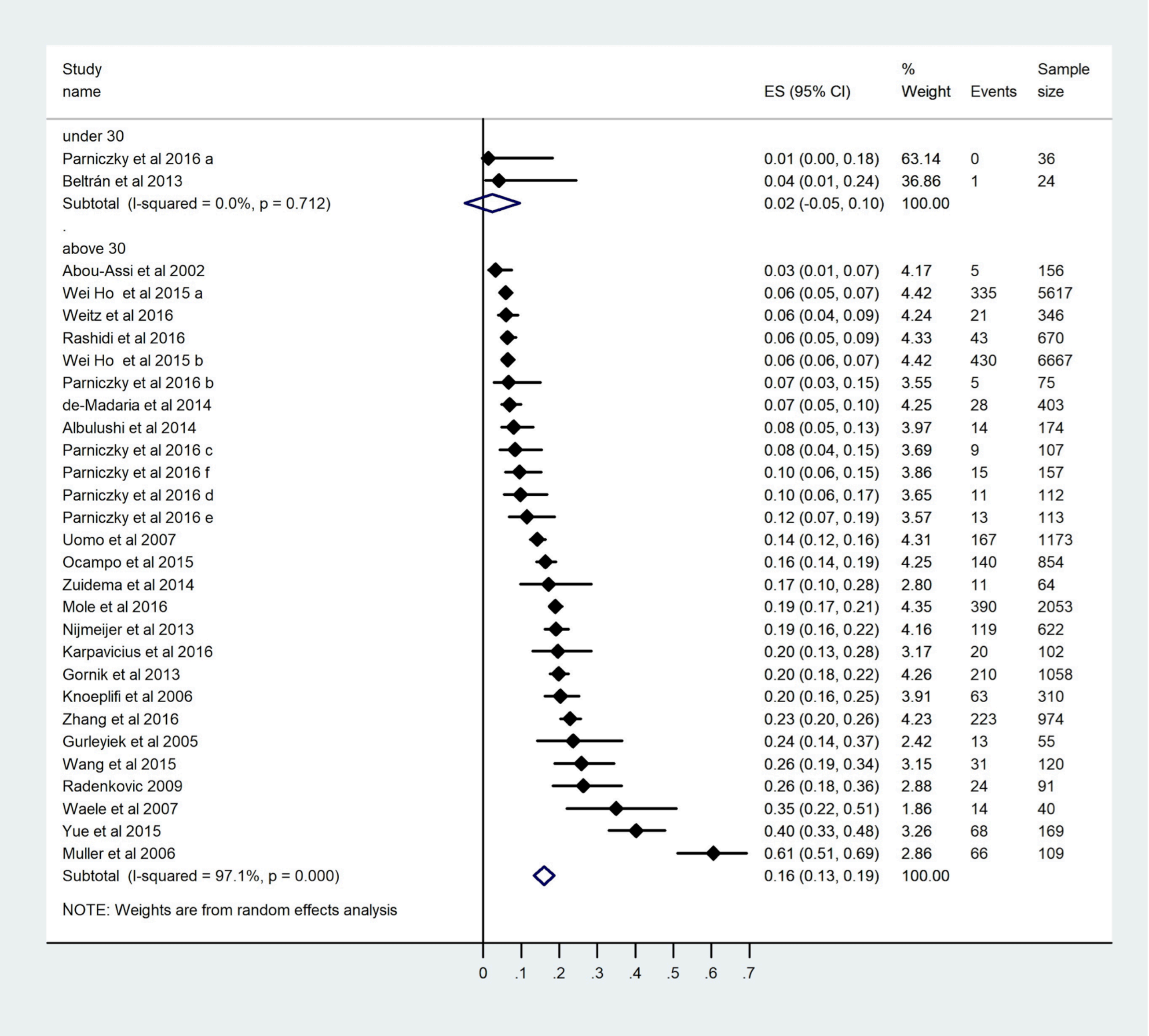

Suppl. Fig. 4. Forest plot of studies evaluating severity at age U30 compared to A30. Full diamonds show the weighted event rates for studies respectively, line represents the 95% confidence interval (CI), and empty diamonds show the pooled results of severe cases. A non-significant difference can be observed in severity under 30 and above 30 (p=0.036).

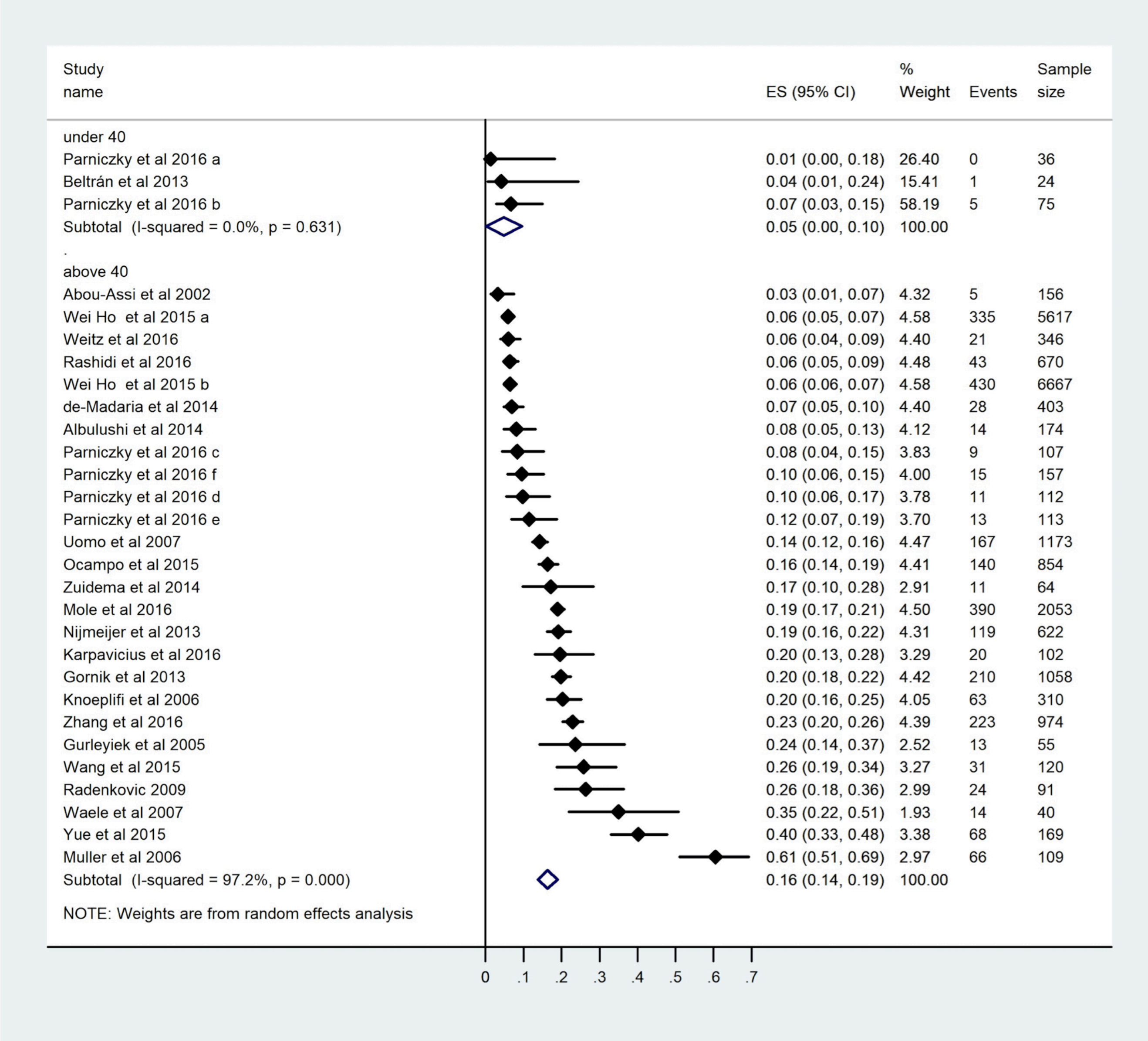

Suppl. Fig. 5. Forest plot of studies evaluating severity at age U40 compared to A40. Full diamonds show the weighted event rates for studies respectively, line represents the 95% confidence interval (CI), and empty diamonds show the pooled results of severe cases A significant difference can be observed in severity under 40 and above 40 (p=0.009).

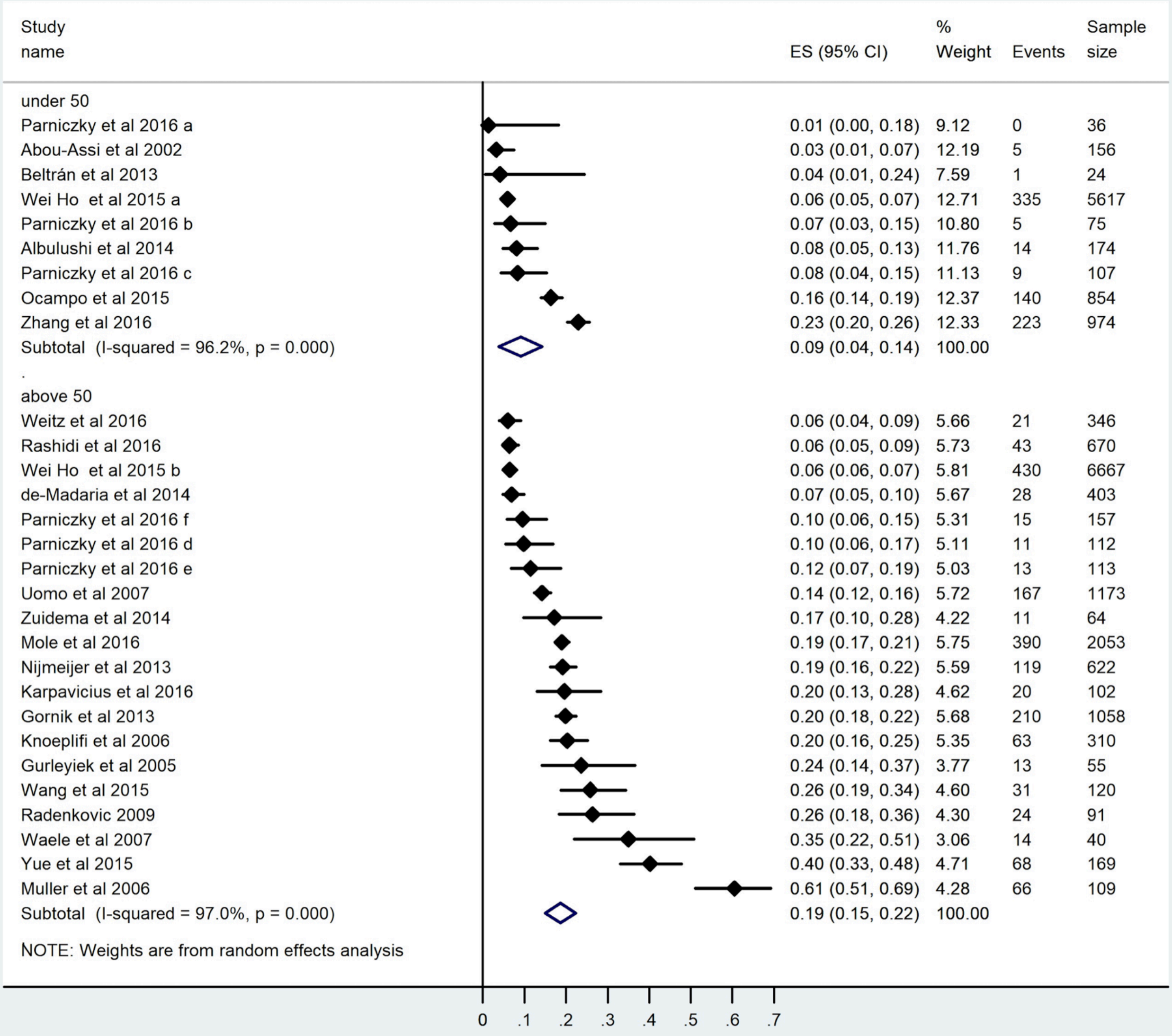

Suppl. Fig. 6. Forest plot of studies evaluating severity at age U50 compared to A50. Full diamonds show the weighted event rates for studies respectively, line represents the 95% confidence interval (CI), and empty diamonds show the pooled results of severe cases. A significant difference can be observed in severity under 50 and above 50 (p=0.021).

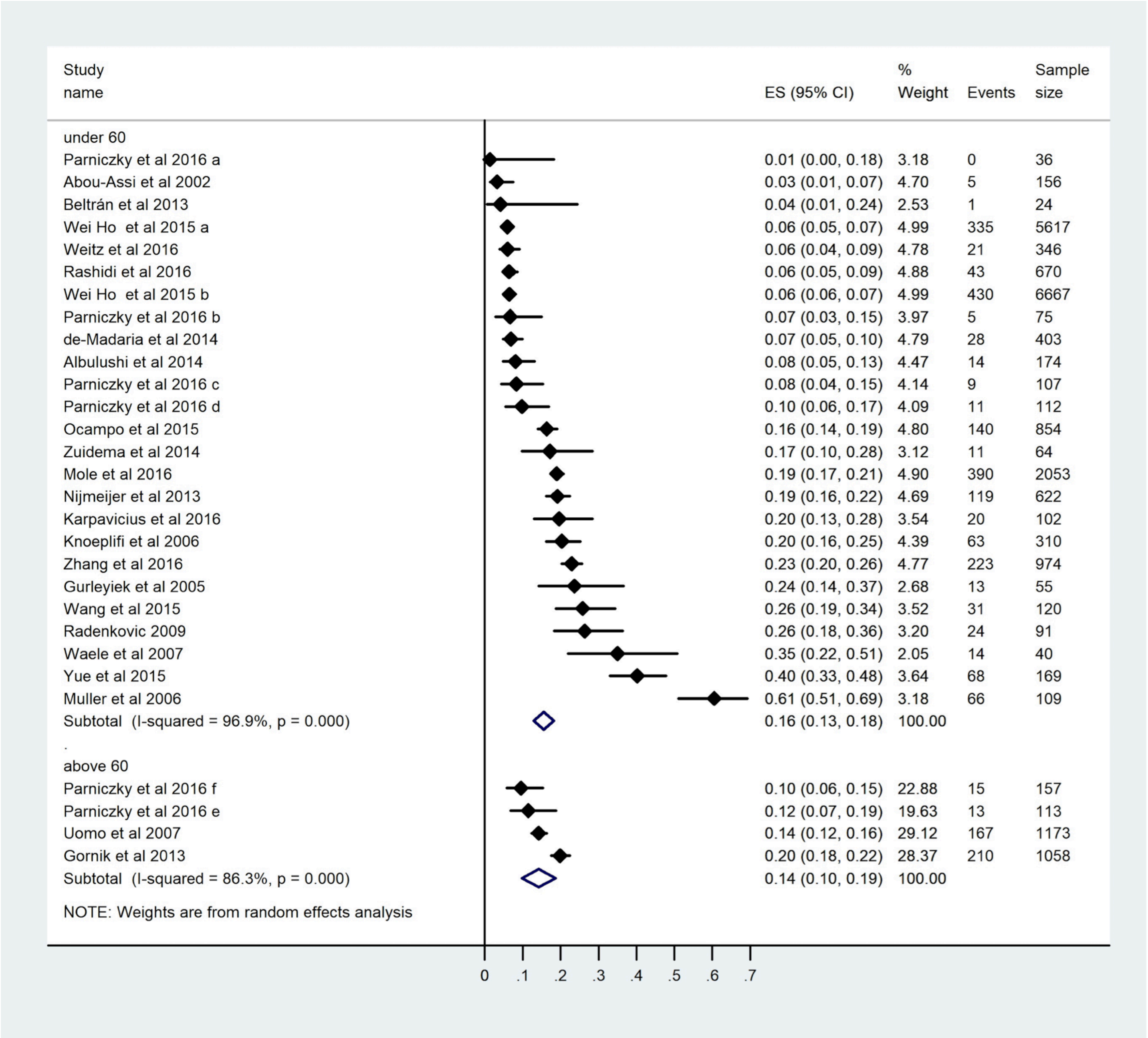

Suppl. Fig. 7. Forest plot of studies evaluating severity at age U60 compared to A60. Full diamonds show the weighted event rates for studies respectively, line represents the 95% confidence interval (CI), and empty diamonds show the pooled results of severe cases. A non-significant difference can be observed in severity under 60 and above 60 (p=0.994).

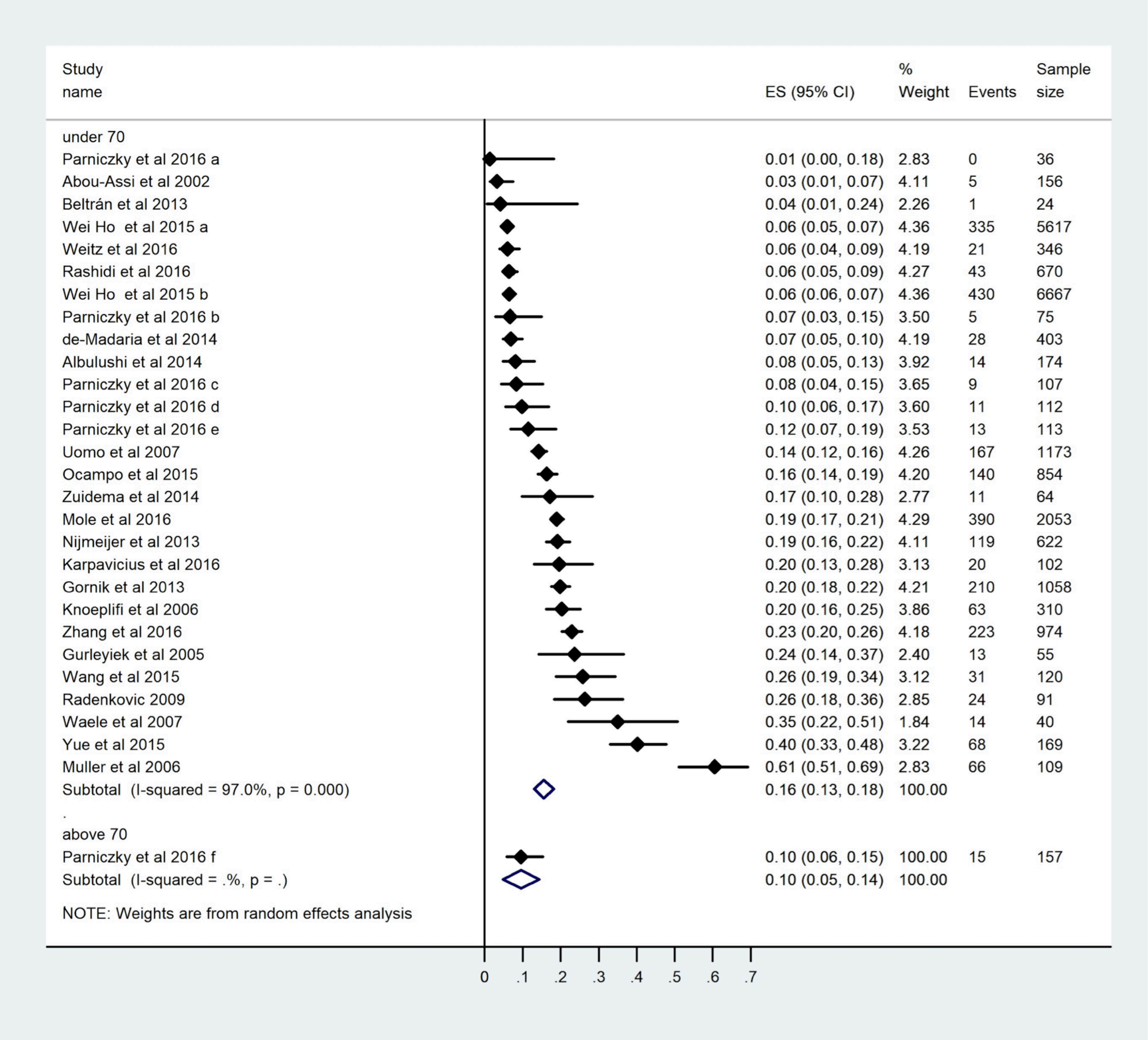

Suppl. Fig. 8. Forest plot of studies evaluating severity at age U70 compared to A70. Full diamonds show the weighted event rates for studies respectively, line represents the 95% confidence interval (CI), and empty diamonds show the pooled results of severe cases. A non-significant difference can be observed in severity under 70 and above 70 (p=0.133).

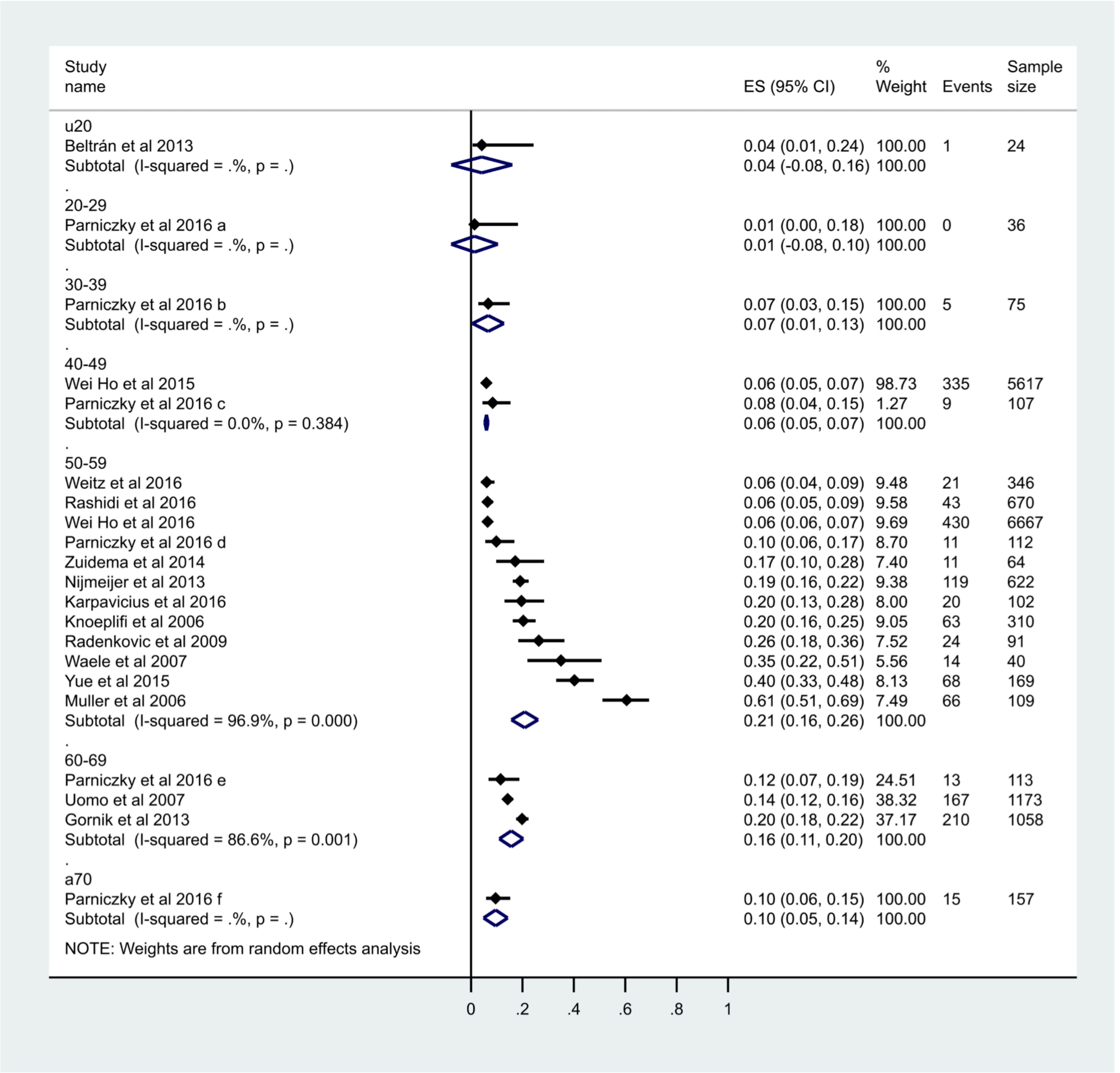

Suppl. Fig. 9. Forest plot of studies to decrease the heterogeneity. Articles where severity was assessed by the Atlanta or the revised Atlanta classification. Full diamonds show the weighted event rates for studies respectively, line represents the 95% confidence interval (CI), and empty diamonds show the pooled results of severe cases. Heterogeneity; I^2= 40-49: 0%, 50-59:96.9%, 60-69:86.6%.

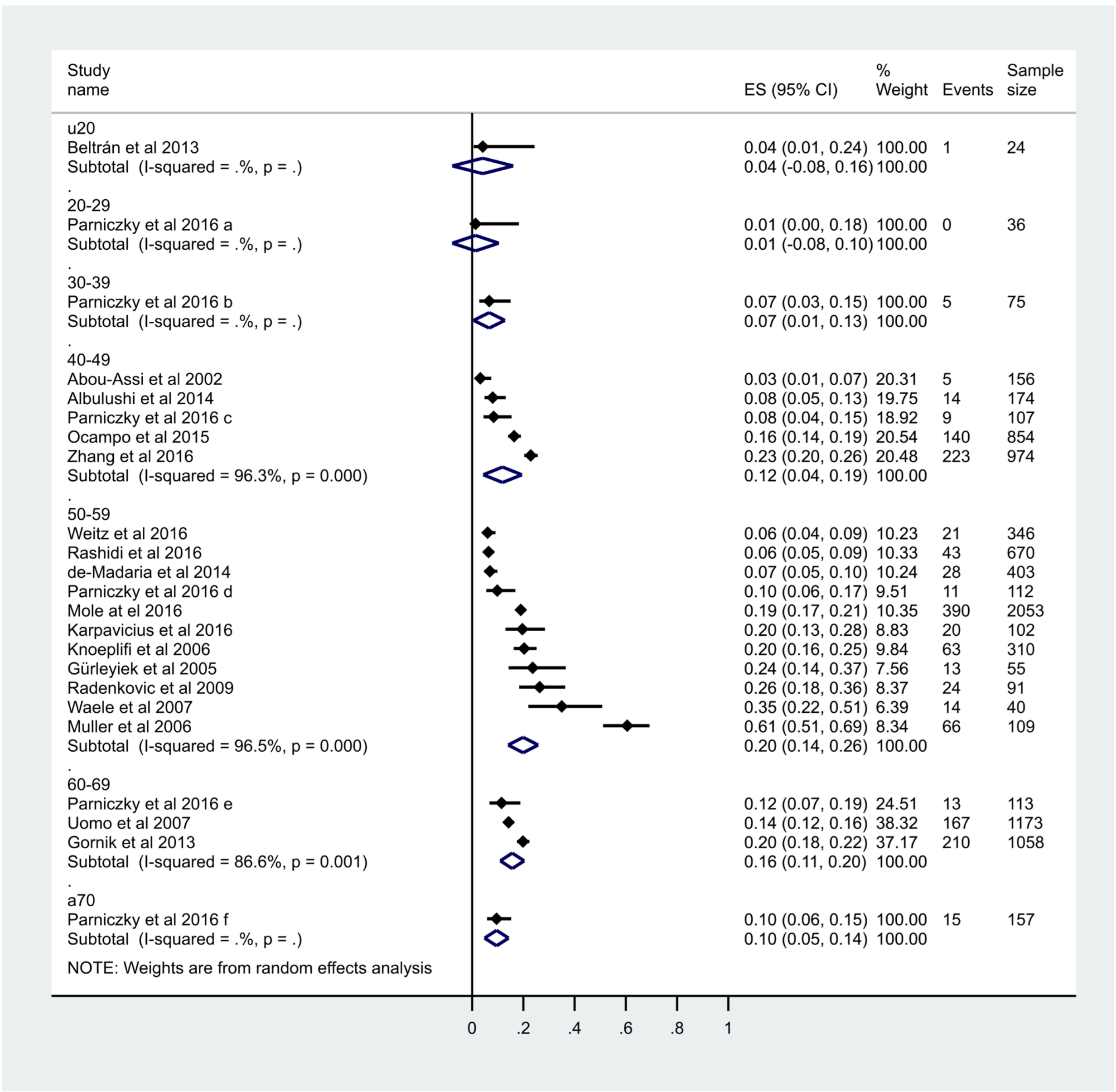

Suppl. Fig. 10. Forest plot of studies to decrease the heterogeneity. Analysis of high quality (NOS 4 and 5) studies. Full diamonds show the weighted event rates for studies respectively, line represents the 95% confidence interval (CI), and empty diamonds show the pooled results of severe cases. Heterogenity; I^2= 40-49: 96.3%, 50-59:96.5%, 60-69:86.6%.

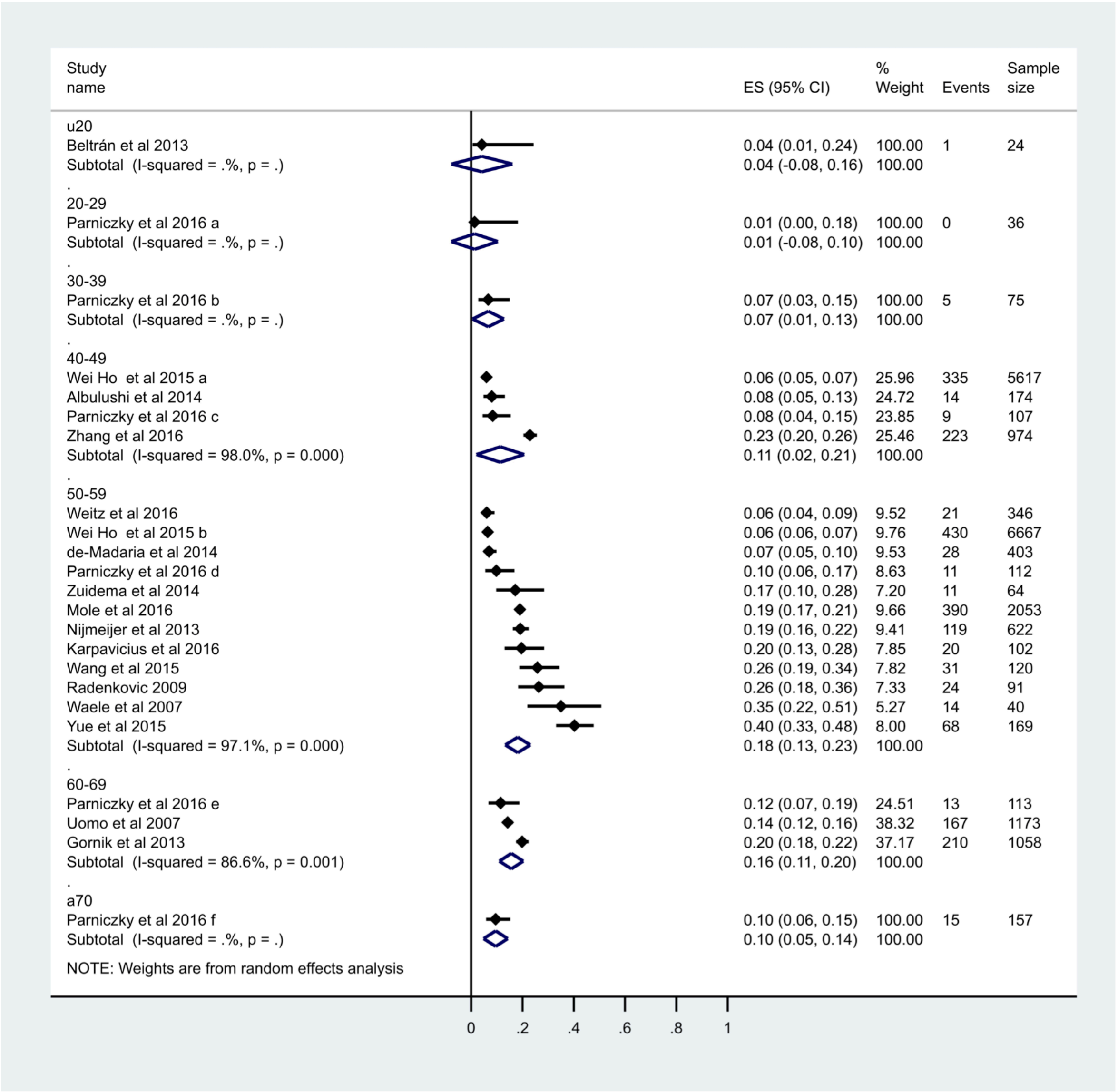

Suppl. Fig. 11. Forest plot of studies to decrease the heterogeneity. Analysis of where age ranges surly not overlap between the groups because of given age ranges concerning severity. Full diamonds show the weighted event rates for studies respectively, line represents the 95% confidence interval (CI), and empty diamonds show the pooled results of severe cases. Heterogenity; I^2= 40-49: 98%, 50-59:97.1%, 60-69:86.6%.

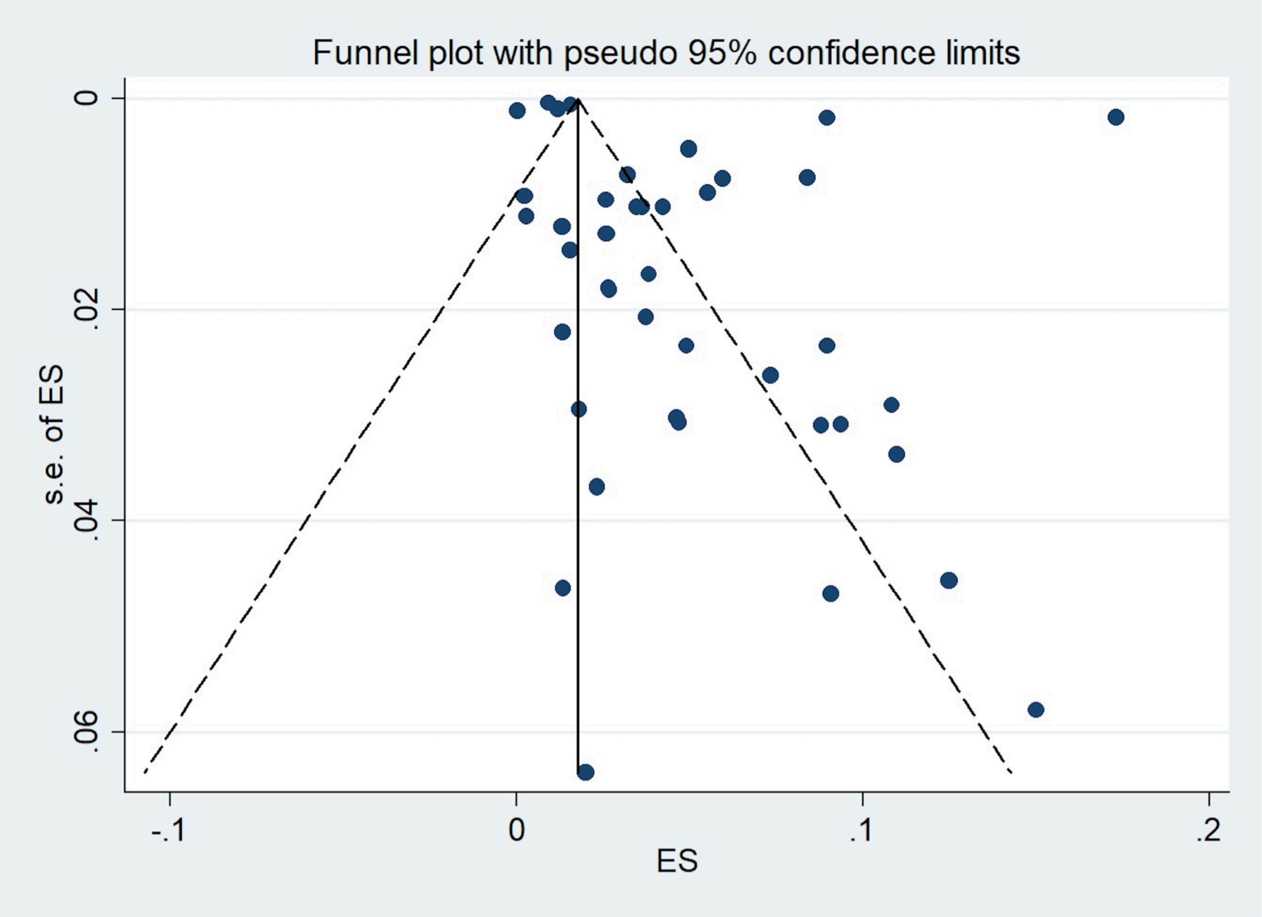

Suppl. Fig. 12. Funnel plot of mortality in terms of small study effect. Funnel plots represent the standard error (SE) plotted against event rates (ES) for each study. The dotted line shows the 95% confidence limits. Plots are slightly on the right side a mild asymmetry, but based on Egger's test publication bias was unlikely(CI: -0.901–9.234; p=0.104).

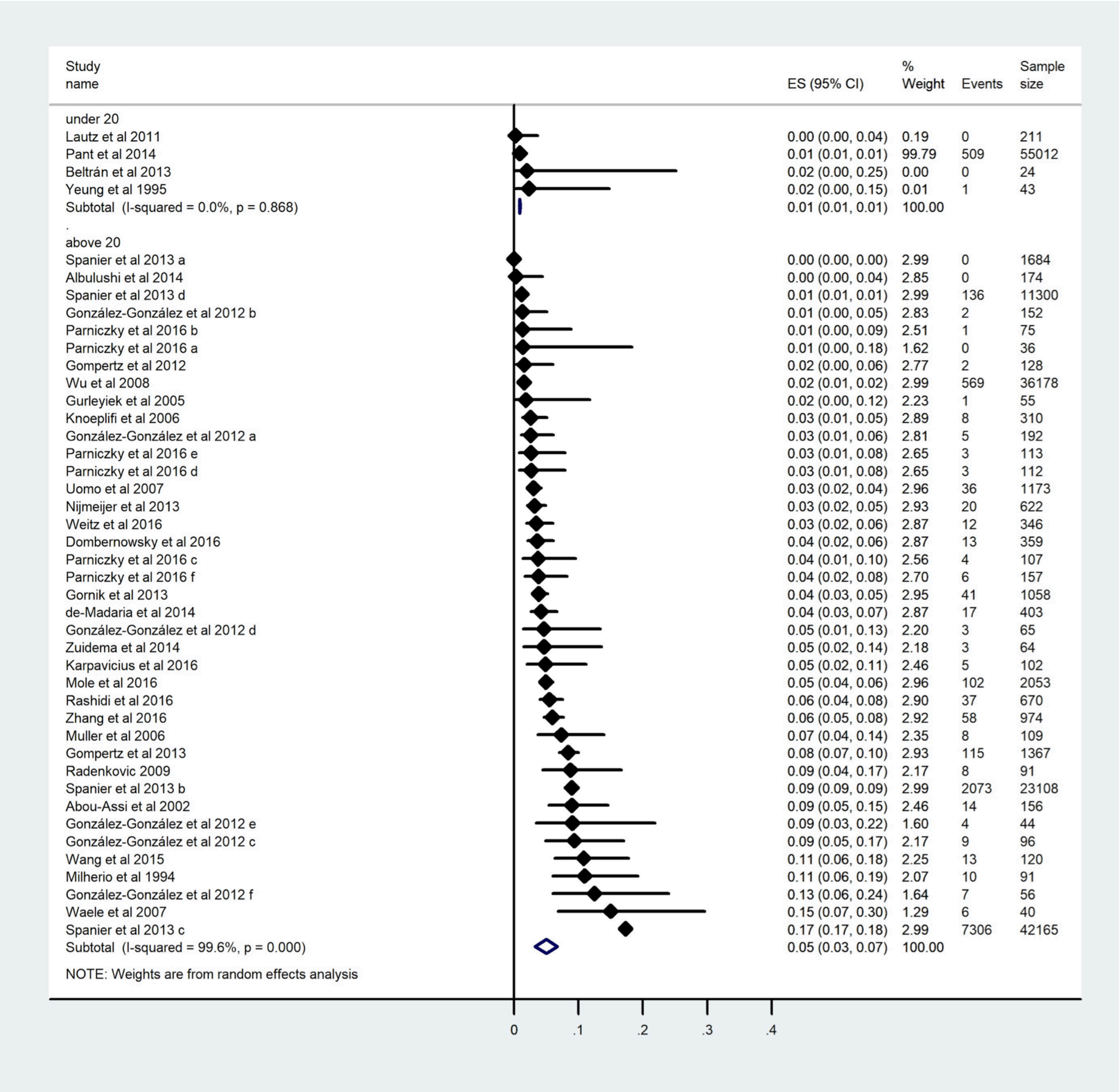

Suppl. Fig. 13. Forest plot of studies evaluating mortality at age U20 compared to A20. Full diamonds show the weighted event rates for studies respectively, line represents the 95% confidence interval (CI), and empty diamonds show the pooled results of severe cases. A significant difference can be observed in mortality under 20 and above 20 ($p<0.001$).

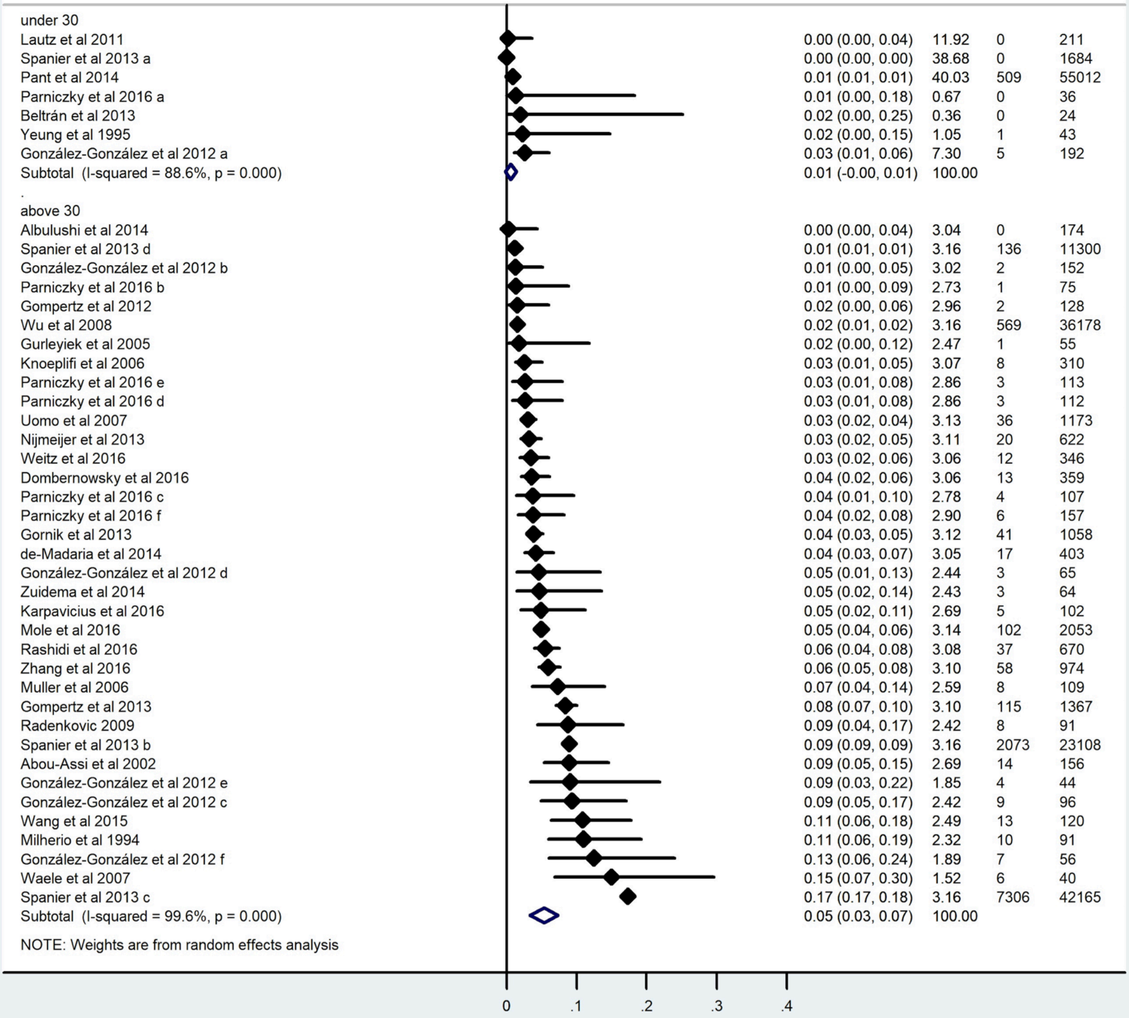

Study name	ES (95% CI)	% Weight	Events	Sample size
under 30				
Lautz et al 2011	0.00 (0.00, 0.04)	11.92	0	211
Spanier et al 2013 a	0.00 (0.00, 0.00)	38.68	0	1684
Pant et al 2014	0.01 (0.01, 0.01)	40.03	509	55012
Parniczky et al 2016 a	0.01 (0.00, 0.18)	0.67	0	36
Beltrán et al 2013	0.02 (0.00, 0.25)	0.36	0	24
Yeung et al 1995	0.02 (0.00, 0.15)	1.05	1	43
González-González et al 2012 a	0.03 (0.01, 0.06)	7.30	5	192
Subtotal (I-squared = 88.6%, p = 0.000)	0.01 (-0.00, 0.01)	100.00		
.				
above 30				
Albulushi et al 2014	0.00 (0.00, 0.04)	3.04	0	174
Spanier et al 2013 d	0.01 (0.01, 0.01)	3.16	136	11300
González-González et al 2012 b	0.01 (0.00, 0.05)	3.02	2	152
Parniczky et al 2016 b	0.01 (0.00, 0.09)	2.73	1	75
Gompertz et al 2012	0.02 (0.00, 0.06)	2.96	2	128
Wu et al 2008	0.02 (0.01, 0.02)	3.16	569	36178
Gurleyiek et al 2005	0.02 (0.00, 0.12)	2.47	1	55
Knoeplifi et al 2006	0.03 (0.01, 0.05)	3.07	8	310
Parniczky et al 2016 e	0.03 (0.01, 0.08)	2.86	3	113
Parniczky et al 2016 d	0.03 (0.01, 0.08)	2.86	3	112
Uomo et al 2007	0.03 (0.02, 0.04)	3.13	36	1173
Nijmeijer et al 2013	0.03 (0.02, 0.05)	3.11	20	622
Weitz et al 2016	0.03 (0.02, 0.06)	3.06	12	346
Dombernowsky et al 2016	0.04 (0.02, 0.06)	3.06	13	359
Parniczky et al 2016 c	0.04 (0.01, 0.10)	2.78	4	107
Parniczky et al 2016 f	0.04 (0.02, 0.08)	2.90	6	157
Gornik et al 2013	0.04 (0.03, 0.05)	3.12	41	1058
de-Madaria et al 2014	0.04 (0.03, 0.07)	3.05	17	403
González-González et al 2012 d	0.05 (0.01, 0.13)	2.44	3	65
Zuidema et al 2014	0.05 (0.02, 0.14)	2.43	3	64
Karpavicius et al 2016	0.05 (0.02, 0.11)	2.69	5	102
Mole et al 2016	0.05 (0.04, 0.06)	3.14	102	2053
Rashidi et al 2016	0.06 (0.04, 0.08)	3.08	37	670
Zhang et al 2016	0.06 (0.05, 0.08)	3.10	58	974
Muller et al 2006	0.07 (0.04, 0.14)	2.59	8	109
Gompertz et al 2013	0.08 (0.07, 0.10)	3.10	115	1367
Radenkovic 2009	0.09 (0.04, 0.17)	2.42	8	91
Spanier et al 2013 b	0.09 (0.09, 0.09)	3.16	2073	23108
Abou-Assi et al 2002	0.09 (0.05, 0.15)	2.69	14	156
González-González et al 2012 e	0.09 (0.03, 0.22)	1.85	4	44
González-González et al 2012 c	0.09 (0.05, 0.17)	2.42	9	96
Wang et al 2015	0.11 (0.06, 0.18)	2.49	13	120
Milherio et al 1994	0.11 (0.06, 0.19)	2.32	10	91
González-González et al 2012 f	0.13 (0.06, 0.24)	1.89	7	56
Waele et al 2007	0.15 (0.07, 0.30)	1.52	6	40
Spanier et al 2013 c	0.17 (0.17, 0.18)	3.16	7306	42165
Subtotal (I-squared = 99.6%, p = 0.000)	0.05 (0.03, 0.07)	100.00		

NOTE: Weights are from random effects analysis

Suppl. Fig. 14. Forest plot of studies evaluating mortality at age U30 compared to A30. Full diamonds show the weighted event rates for studies respectively, line represents the 95% confidence interval (CI), and empty diamonds show the pooled results of severe cases. A significant difference can be observed in mortality under 30 and above 30 (p=0.001).
