# Supplementary material for: Aging and Comorbidities in Acute Pancreatitis I: A Meta-Analysis and Systematic Review Based on 194,702 Patients
Source: Front Physiol. 2019 Apr 2;10:328. doi: 10.3389/fphys.2019.00328 (PMC6454469; doi:10.3389/fphys.2019.00328)
Supplement: Supplementary file 2 [file Data_Sheet_2.pdf]

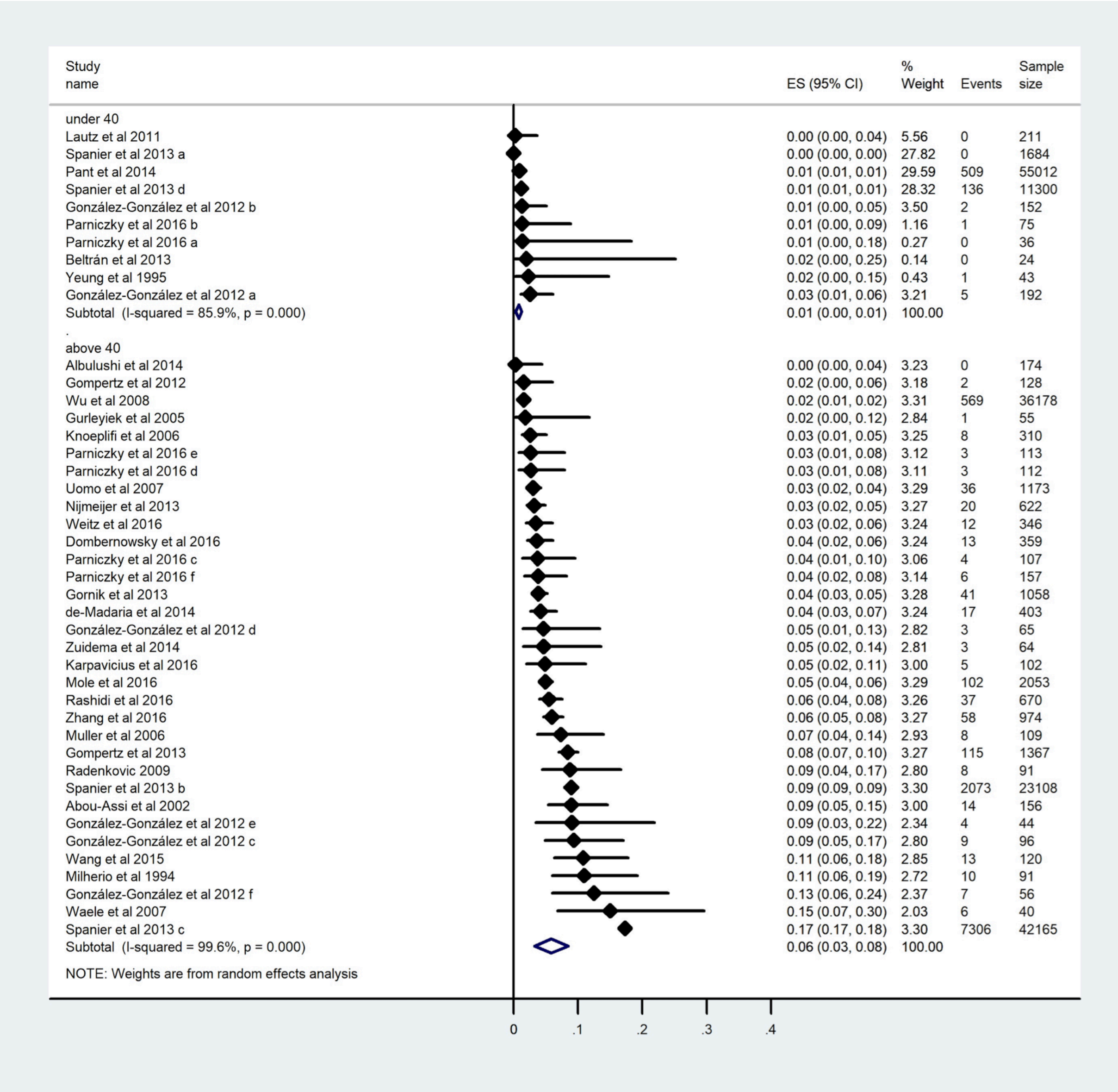

Suppl. Fig. 15. Forest plot of studies evaluating mortality at age U40 compared to A40. Full diamonds show the weighted event rates for studies respectively, line represents the 95% confidence interval (CI), and empty diamonds show the pooled results of severe cases. A significant difference can be observed in mortality under 40 and above 40 ($p<0.001$).

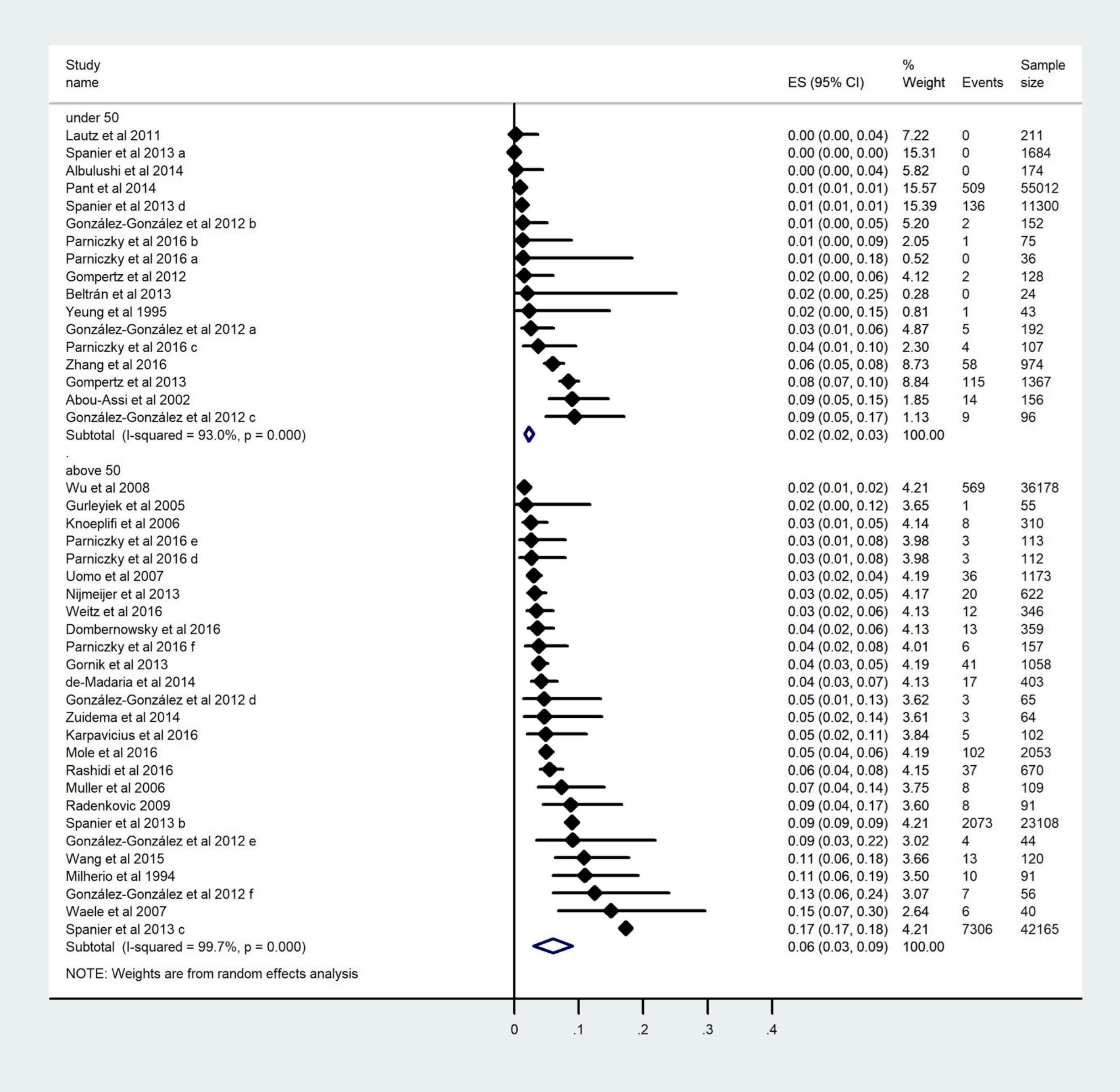

Suppl. Fig. 16. Forest plot of studies evaluating mortality at age U50 compared to A50. Full diamonds show the weighted event rates for studies respectively, line represents the 95% confidence interval (CI), and empty diamonds show the pooled results of severe cases. A significant difference can be observed in mortality under 50 and above 50 (p=0.018).

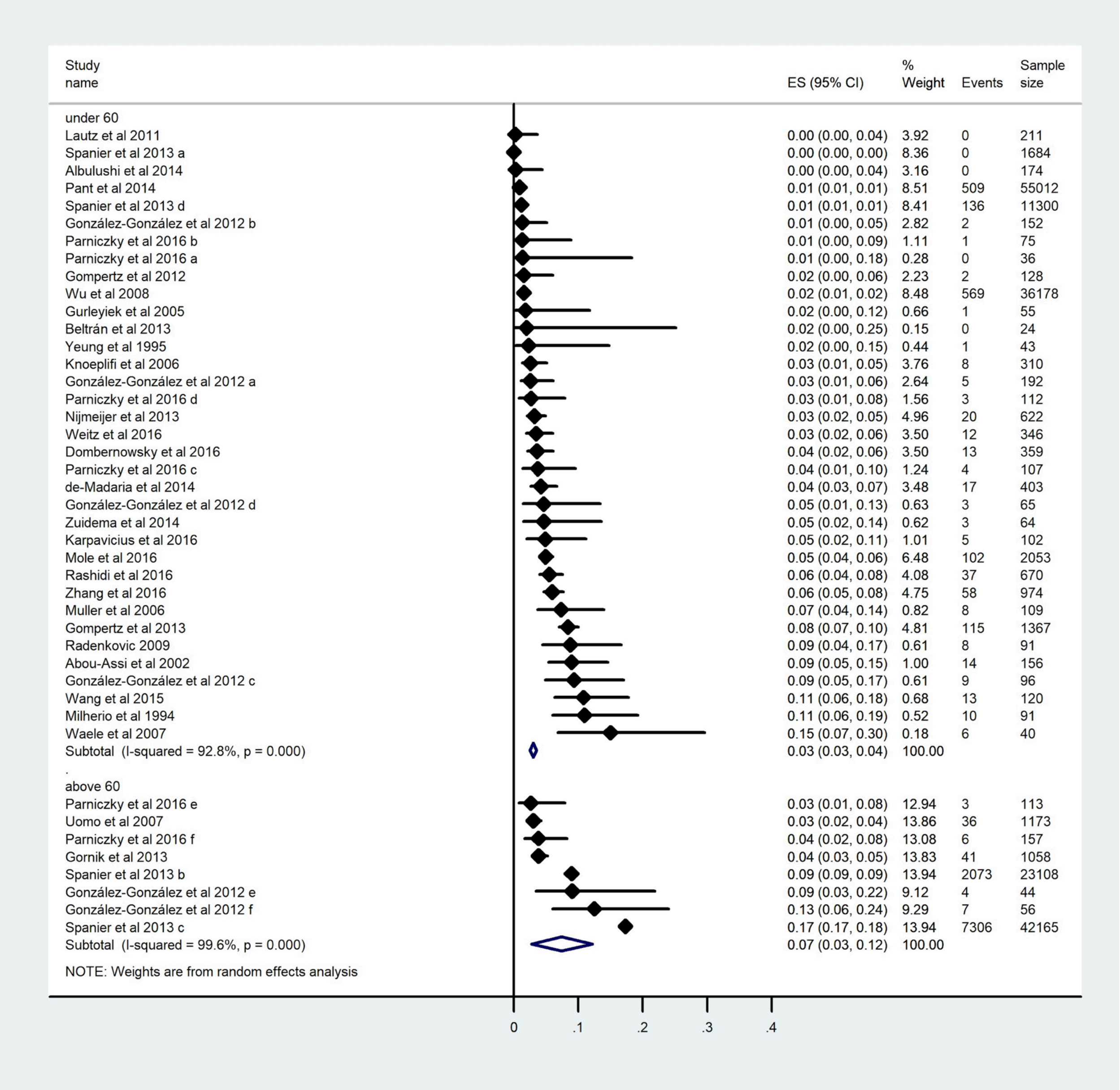

Suppl. Fig. 17. Forest plot of studies evaluating mortality at age U60 compared to A60. Full diamonds show the weighted event rates for studies respectively, line represents the 95% confidence interval (CI), and empty diamonds show the pooled results of severe cases. A significant difference can be observed in mortality under 60 and above 60 (p=0.028).

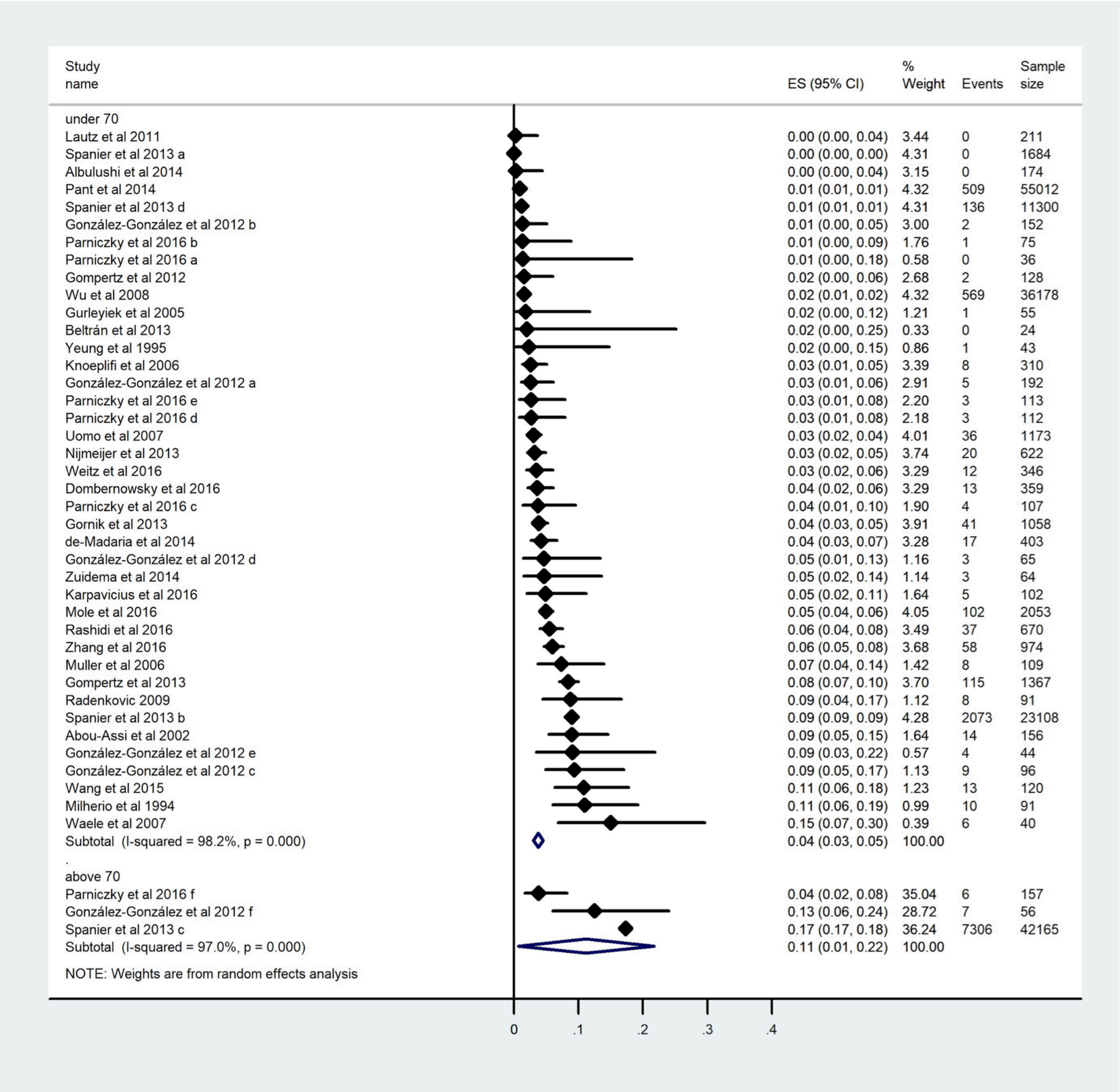

Suppl. Fig. 18. Forest plot of studies evaluating mortality at age U70 compared to A70. Full diamonds show the weighted event rates for studies respectively, line represents the 95% confidence interval (CI), and empty diamonds show the pooled results of severe cases. A significant difference can be observed in mortality under 70 and above 70 (p=0.038).

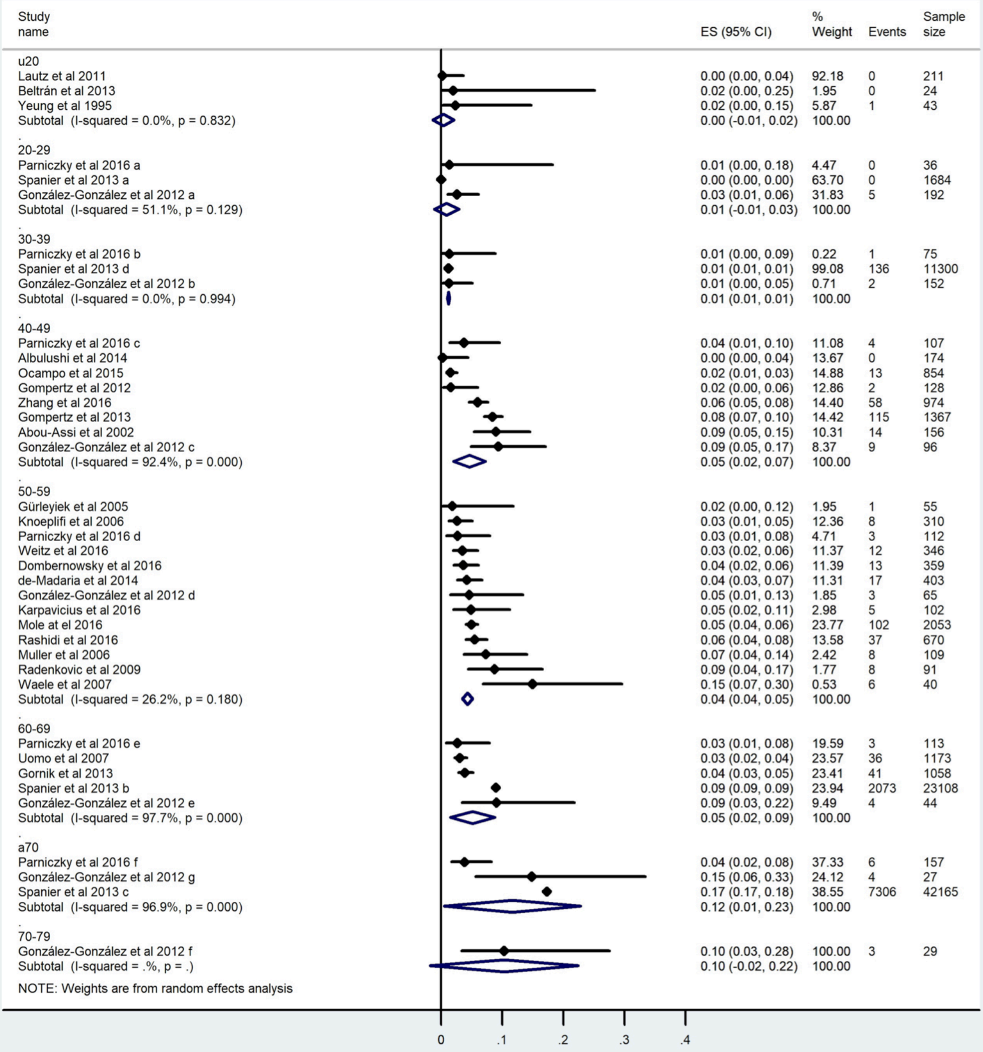

Suppl. Fig. 19. Suppl. Fig. 10. Forest plot of studies to decrease the heterogeneity. Analysis of high quality (NOS 4 and 5) studies concerning mortality. Full diamonds show the weighted event rates for studies respectively, line represents the 95% confidence interval (CI), and empty diamonds show the pooled results of severe cases. Heterogenity; I^2= 40-49: 96.3%, 50-59:96.5%, 60-69:86.6%.
